# Antioxidant Activity and Inhibition of Liver Cancer Cells’ Growth of Extracts from 14 Marine Macroalgae Species of the Mediterranean Sea

**DOI:** 10.3390/foods12061310

**Published:** 2023-03-19

**Authors:** Nikolaos Goutzourelas, Dimitrios Phaedon Kevrekidis, Sofia Barda, Paraskevi Malea, Varvara Trachana, Stavroula Savvidi, Alkistis Kevrekidou, Andreana N. Assimopoulou, Andreas Goutas, Ming Liu, Xiukun Lin, Nikolaos Kollatos, Grigorios D. Amoutzias, Dimitrios Stagos

**Affiliations:** 1 Department of Biochemistry and Biotechnology, School of Health Sciences, University of Thessaly, Biopolis, 41500 Larissa, Greece; 2 Laboratory of Forensic Medicine and Toxicology, Department of Medicine, Aristotle University of Thessaloniki, 54124 Thessaloniki, Greece; 3 Department of Botany, School of Biology, Aristotle University of Thessaloniki, 54124 Thessaloniki, Greece; 4 Department of Biology, Faculty of Medicine, University of Thessaly, Biopolis, 41500 Larissa, Greece; 5 Laboratory of Organic Chemistry, School of Chemical Engineering, Aristotle University of Thessaloniki, 54124 Thessaloniki, Greece; 6 Environmental Engineering Laboratory, Department of Chemical Engineering, Aristotle University of Thessaloniki, 54124 Thessaloniki, Greece; 7 Key Laboratory of Marine Drugs, Ministry of Education, School of Medicine and Pharmacy, Ocean University of China, Qingdao 266003, China; 8 Laboratory for Marine Drugs and Bioproducts of Qingdao National Laboratory for Marine Science and Technology, Qingdao 266237, China; 9 Department of Pharmacology, School of Pharmacy, Southwest Medical University, 319 Zhongshan Road, Luzhou 646000, China

**Keywords:** marine macroalgae, seaweeds, antioxidant, chemoprevention, anticancer, DNA damage, liver cancer, polyphenols, Aegean Sea

## Abstract

Macroalgae exhibit beneficial bioactivities for human health. Thus, the aim of the present study was to examine the antioxidant and anticancer potential of 14 macroalgae species’ extracts, namely, *Gigartina pistillata*, *Gigartina teedei*, *Gracilaria gracilis*, *Gracilaria* sp., *Gracilaria bursa pastoris*, *Colpomenia sinuosa*, *Cystoseira amentacea*, *Cystoseira barbata*, *Cystoseira compressa*, *Sargassum vulgare*, *Padina pavonica*, *Codium fragile*, *Ulva intestinalis*, and *Ulva rigida*, from the Aegean Sea, Greece. The antioxidant activity was assessed using DPPH, ABTS^•+^, ^•^OH, and O_2_^•−^ radicals’ scavenging assays, reducing power (RP), and protection from ROO^•^-induced DNA plasmid damage assays. Moreover, macroalgae extracts’ total polyphenol contents (TPCs) were assessed. Extracts’ inhibition against liver HepG2 cancer cell growth was assessed using the XTT assay. The results showed that *G. teedei* extract’s IC_50_ was the lowest in DPPH (0.31 ± 0.006 mg/mL), ABTS^•+^ (0.02 ± 0.001 mg/mL), ^•^OH (0.10 ± 0.007 mg/mL), O_2_^•−^ (0.05 ± 0.003 mg/mL), and DNA plasmid breakage (0.038 ± 0.002 mg/mL) and exhibited the highest RP (RP_0.5AU_ 0.24 ± 0.019 mg/mL) and TPC (12.53 ± 0.88 mg GAE/g dw). There was also a significant correlation between antioxidant activity and TPC. *P. pavonica* (IC_50_ 0.93 ± 0.006 mg/mL) exhibited the highest inhibition against HepG2 cell growth. Conclusively, some of the tested extracts exhibited significant chemopreventive properties, and so they may be used for food products.

## 1. Introduction

Chemoprevention is currently considered among the most important strategies for fighting cancer [1]. Specifically, chemoprevention is defined as the use of natural or synthetic compounds as drugs or through the diet for the prevention or even the reversal of carcinogenesis [2]. One of the cancer types suggested as suitable for the application of chemoprevention is liver cancer [1]. Liver cancer is the fifth most frequent tumor type worldwide and the third in terms of mortality [1]. So far, there has not been an effective conventional therapy for liver cancer. Surgical resection, local ablation, and liver transplantation are the most common treatments for a small proportion of patients with early-stage hepatocellular carcinoma [1,3]. The main drug used for the treatment of advanced liver cancer is sorafenib [3]. Sorafenib inhibits cancer cell proliferation and tumor angiogenesis through the inhibition of several serine/threonine kinases such as Raf-1, and receptor tyrosine kinases such as vascular endothelial growth factor receptors (VEGFRs) and platelet-derived growth factor (PDGF) [3]. However, sorafenib provides modest gains in survival [1,3]. Thus, there is a great need for alternative treatments for use as chemopreventive agents [1,3]. Epidemiological studies have shown that populations of many countries with high consumption of fish and seafood have low prevalence of particular type of cancers (e.g., lung, breast, colorectal, and prostate cancers) [4]. This observation has led to extensive investigations of the benefits of compounds present in edible marine organisms, including marine macroalgae, as cancer chemopreventive agents [4].

Numerous studies have demonstrated the association between oxidative stress (i.e., the overproduction of free radicals) and cancer in humans [5]. Thus, the uptake of antioxidants through the diet or as food supplements has been suggested for cancer prevention [1]. Interestingly, compounds such as polyphenols and bromophenols isolated from marine macroalgae have been shown by our research group and others in in vitro and in vivo studies to possess antioxidant and anticancer activities [6,7,8]. For example, macroalgae’s compounds have been shown to act as reactive oxygen species’ (ROS) scavengers, and consequently they may provide protection from ROS-induced DNA damage in non-cancerous cells, a major cause for the initiation of carcinogenesis [6,8]. Although marine macroalgae exhibit great interest because of their bioactive properties for human health, they are considered as an underexploited resource [9,10].

Macroalgae (also known as seaweeds) along with seagrasses are the main primary producers and have an essential role in the structure and function of coastal and estuarine environments [11,12]. They are characterized by the formation of productive communities with great biodiversity [12]. Macroalgae are used in various applications such as environmental indicators of water quality, feasible alternatives to fossil fuels, and fertilizers [13,14]. In addition, there is currently great research interest for macroalgae as an important source for human nutrition [15]. Microalgae contain a variety of cellular components such as proteins, cellulose, polysaccharides, minerals, and phenolic compounds, which exhibit beneficial properties for human health such as antioxidant, anticancer, antibacterial, antimicrobial, antifungal, and antihypertensive [13]. The interspecific variation in macroalgal biochemical composition is expected, as macroalgae belong to different phylogenetic groups (i.e., Phaeophyceae, Rhodopyta, Chlorophyta), as well as functional form groups (i.e., filamentous, coarsely-branch, sheet, thick-leathery), which determine their physiological processes [13,16,17,18]. Additionally, as macroalgae grow worldwide, they are exposed to various abiotic and biotic environmental stresses that stimulate the production of bioactive components such as polyphenols, fatty acids, sterols, and carbohydrates [13,17,18].

In the Mediterranean Sea, which is characterized by high biodiversity, 1351 taxa of benthic macroalgae have been recorded, corresponding to 16.2% of all macroalgae worldwide [19]. The Greek coasts, a major part of the Eastern Mediterranean Sea, are inhabited by flora species belonging to different geographic affinities (e.g., endemic, eastern Atlantic temperate, Indopacific tropical) [20]. In addition, the Greek coasts along with Turkish coasts present the highest macroalgal biodiversity in the Eastern Mediterranean Sea due mainly to different oceanographic or geomorphological characteristics of coastal waters [21]. The taxonomical group of red algae dominates in terms of diversity in the macroalgae found in Greek coasts [22]. The Greek macroalgae include in total about 550 taxa [23,24]. Especially, in Greek coasts, benthic macroalgal species are more frequently found in the North and South Aegean Sea and in shallow sheltered body types (10–45 species/0.04 m^2^) [23,24]. The Aegean Sea is an elongated embayment of the Mediterranean Sea and covers an area of about 215,000 km^2^. Since the Aegean Sea has great biodiversity of marine organisms including endemic and rare species, it is considered an important area to study marine resources [25]. For example, Montalvao et al. [25] examined 72 macroalgae species collected from the Turkish coast of the Aegean Sea for their inhibitory activity against growth of prostate and breast cancer cells. They found that the most potent species were *Cystoseira barbata*, *Cystoseira crinita*, *Cystoseira stricta*, *Dictyopteris membranacea*, *Hypnea musciformis*, *Laurencia papilossa*, and *Sargassum vulgare* [25]. In another study [26], the antioxidant activity of five brown macroalgae species from the Aegean Sea (Izmir coast, Turkey) was examined. Moreover, Guner et al. [27] examined the antioxidant activity and cytotoxicity against liver cancer cells of *Cystoseira compressa* collected from the Turkish Coast of Urla in the Aegean Sea. In these studies, the main metabolites found in macroalgae were polyphenols, phenols, terpenes, hydrocarbons, and aldehydes [26,27]. However, since only a few studies on the antioxidant and anticancer compounds of macroalgae from the Aegean Sea have been conducted so far, more research is needed. 

Thus, the aim of the present study was to investigate the chemopreventive potential (i.e., antioxidant activity and inhibition of cancer cell growth) of extracts of fourteen marine macroalgae species collected from the Northern Aegean Sea, Greece. Specifically, the most abundant and dominant seaweed species in the collection area at the sampling period were examined. The collection of abundant species was also necessary, since significant extracts’ amounts were required to perform all the assays. The collected species belonged, for comparison reasons, to all three phylogenetic (i.e., Chlorophyta, Phaeophyceae, and Rhodophyta) and functional form groups of macroalgae. It should be noted that most of these species (e.g., *Ulva rigida*, *Ulva intestinalis*, *Codium fragile*, *Gracilaria gracilis*, *G. bursa pastoris*, *Gracilaria* sp., *Cystoseira barbata*, and *Padina pavonia*) were also recorded during other sampling periods from our research group in the collection area [16,17,28,29]. The antioxidant activity was assessed in vitro using free radical scavenging, reducing power (RP), and protection from ROS-induced DNA plasmid breakage assays. In addition, macroalgae extracts’ inhibitory activity against liver cancer cell growth was assessed. Moreover, macroalgae extracts’ total polyphenolic content (TPC) values were evaluated. Correlation analysis was also performed between the different bioactivities and TPC values. Most of the tested macroalgae species collected from the Aegean Sea have never been investigated previously for their chemopreventive activities. 

## 2. Materials and Methods

### 2.1. Marine Macroalgae Species Collection

**Figure 1:**
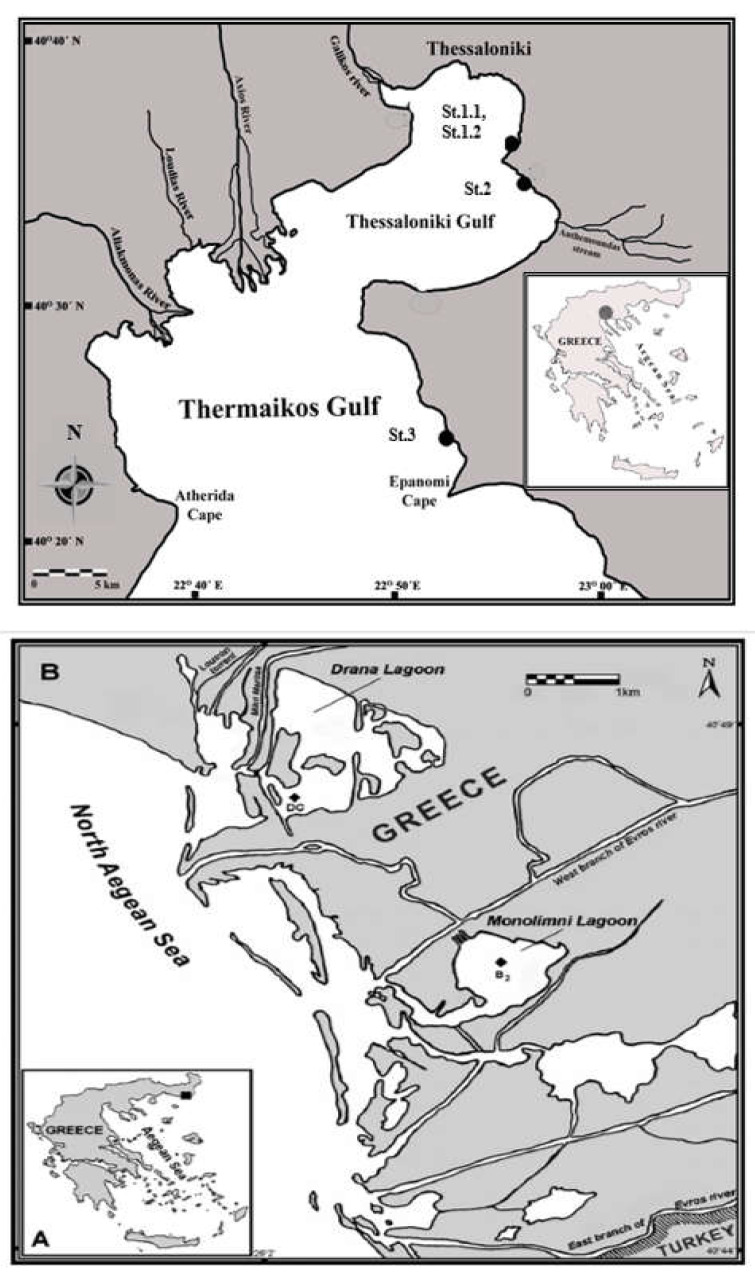
The above map shows Thermaikos Gulf and indicates the sampling stations (i.e., St.1, St.2, St.3). The below maps ((**A**): whole map of Greece; (**B**): map of collection region)show Evros Delta and indicate the sampling station (i.e., St.4 Monolimni Lagoon). In above and below maps, the geographical location of the study sites is indicated.

Samples of fourteen dominant marine macroalgae species were collected from June to September 2020 from the Thermaikos Gulf ( Thessaloniki, Greece) and the Monolimni lagoon (Evros River Delta, Greece), Northern Aegean Sea, Mediterranean Sea (Figure 1, Table 1). More specifically, fourteen seaweed species (entire thalli) were collected from four stations, namely, St1 (48°58′97.37″ Ν 22°94′41.42″ Ε, with two substations St1.1 and St1.2), St2 (40°40′64.46″ N, 22°89′34.38″ E), St3 (40°30′12.5″ N 22°51′25.1″ E) of the Gulf of Thessaloniki (Figure 1, Table 1), and St4 (40°45′ N, 26°01′ E) at the outer part of the lagoon of Evros River Delta (Figure 1, Table 1). Rhodophyta (i.e., red macroalgae) were represented by five species, namely, *Gigartina pistillata*, *Gigartina teedei*, *Gracilaria gracilis*, *Gracilaria* sp., and *Gracilaria bursa pastoris* (S.G.Gmelin) P.C. Silva; Phaeophyceae (i.e., brown macroalgae) by six species, namely, *Colpomenia sinuosa*, *Cystoseira amentacea*, *Cystoseira barbata*, *Cystoseira compressa*, *Sargassum vulgare*, and *Padina pavonica*; and Chlorophyta (i.e., green macroalgae) by three taxa, namely, *Codium fragile*, *Ulva intestinalis*, and *Ulva rigida* (Table 1). The nomenclature and classification of organisms were based on the following floral catalogs and studies [23,24,30,31,32,33,34,35,36,37,38,39,40]. Two of these species belonged to the sheet functional group, five species to the coarsely branched group, and seven species to the thick–leathery group (Table 1) [16,17,23,24,30,31,32,33,34,35,36,37,38,39,40,41].

At the sampling stations, the marine macroalgae species were randomly collected by hand, wearing gloves, directly from the substrate, using a spatula, from 50–70 cm of depth. Samples of the same species collected from a common station were pooled, having a total biomass ranging from 300 to 15,000 g wet wt. Then, they were rinsed in seawater and transported to the laboratory in large containers (50 L) with seawater from the collection area. 

In the laboratory, the macrophyte species were identified to the lowest possible taxon [23,24,30,31,32,33,34,35,36,37,38,39,40].

Subsequently, they were washed with double distilled water, and any epiphyte, dead thalli part, and sediment were carefully removed with nylon brushes. They were dried at 50 °C for 48 h in the oven (Friocell, MMM Medcenter Einrichtungen GmbH; Munich, Germany) to constant weight and ground using an agate mill (MixerMill MM200, Retsh; Haan, Germany). 

### 2.2. Extract Preparation

The isolation of the extracts from the marine macroalgae was made according to Farasat et al. [42] with modifications. After grinding, macroalgae were soaked for extraction in 80% methanol solution (1:30 dried weight sample to solvent volume), elaborated with a UP400S Hielscher sonicator (Teltow, Germany) at 20 cycles and 70% amplitude for 20 min, and left in a shaker incubator (Innova^®^ 40, New Brunswick Scientific; St Albans, UK) at 25 °C and 150 rpm for 48 h. Afterwards, the extract solutions were filtered using Whatman filter paper (0.45 μm). The solvent was removed under reduced pressure by a rotary evaporator (IKA, Werke RV-06-ML; Staufen, Germany) at 30 °C and 150 rpm, followed by freeze drying (Coolsafe^TM^, Scanvac; Allerod, Denmark) for 24 h, so as to produce an extract in the form of a powder. The dried powder was weighed to evaluate the percentage yield of the extraction process using the following equation:

Extraction yield (%) = [dry extract (g)/dry seaweed (g)] × 100(1)

The extracts were kept at −20 °C until further use.

### 2.3. Assessment of Macroalgae Extracts’ Polyphenolic Contents

Macroalgae extracts’ TPC values were evaluated spectrophotometrically at 765 nm by using the Folin–Ciocalteu reagent as described previously [43]. TPC was determined by a standard curve of absorbance values in correlation with standard concentrations (50–1500 μg/mL) of gallic acid. The TPC was expressed as mg of gallic acid equivalents (GAE) per g of dry weight (dw) of extract.

Moreover, HPLC-DAD analysis was performed to identify individual polyphenols and simple phenols in macroalgae extracts. Analysis by HPLC was performed on an ECOM analytical HPLC instrument, model ECS05 (Prague, Czech Republic), consisting of a quaternary gradient pump (ECP2010H) and a gradient box with a degasser (ECB2004) coupled with a diode array detector (ECDA2800 UV-Vis PDA Detector). Chromatographic separation of the samples was carried out on a Fortis SpeedCore column (C18, 2.6 um, 100 × 4.6 mm) (Cheshire, United Kingdom). Millipore water acidified with 0.1% formic acid (A) and methanol (B) was utilized as the elution system, with a total flow rate of 1 mL/min. The elution gradient started with 90% A, which remained constant for 5 min, and at 8.5 min it was set to 72% A and at 30 min to 40% A; this remained constant for 3 min. After each injection, the system was equilibrated for 3 min at the initial conditions. The column temperature was set at 25 °C, and the injection volume was 10 μL. The detection of the peaks was performed at 280, 270, 328, and 318 nm. Data were processed by using Clarity Chromatography Software v8.2 (DataApex Ltd., Thessaloniki, Greece) 

For identifying phenolic compounds in macroalgae samples and to later proceed with the quantification, the following mixture of standards was used: caftaric acid, caffeic acid, epigallocatechine gallate, p-coumaric acid, chicoric acid, trans-ferulic acid, quercetin, sinapic acid, rutin, and trans-cinnamic acid (Merck, Darmstadt, Germany). Standards were diluted in methanol and analyzed at 280, 270, 328, and 318 nm. The mixture of standards at a concentration range from 0.78 to 200 ppm was used for the construction of each calibration curve. Analyses of the phenolic contents were carried out in the macroalgae extracts at 7000 ppm concentration in methanol and were identified by the standards. 

### 2.4. DPPH Radical Scavenging Assay

The 2,2-diphenyl-picrylhydrazyl (DPPH^•^) assay was performed as described previously [43]. In brief, different concentrations of macroalgae extract in aqueous solution were added to 1.0 mL of methanolic solution of DPPH^•^ radical (100 μM). Specifically, each macroalgae extract was dissolved in double distilled water to make stock solutions (300 mg/mL). These stocks were used for achieving different extract concentrations by making serial dilutions. One hundred μL was added from each extract concentration to the reaction mixture, having a total volume of 1 mL. After mixed by vortexing, the samples were incubated at room temperature in the dark for 20 min, and the absorbance was measured at 517 nm. The measurement was conducted on a Perkin Elmer Lambda 25 UV/VIS spectrophotometer (Waltham, MA, USA). In each experiment, the tested sample alone in methanol was used as a negative control. These negative controls were used to avoid the possible interference of the extract’s absorbance by itself, with the absorbance measured by the assay. The absorbance of these negative controls was subtracted by the absorbance of the corresponding samples. DPPH^•^ alone in methanol was used as a control. Ascorbic acid was used as a positive control for the antioxidant activity.

The percentage of radical scavenging capacity (RSC) of the tested extracts was calculated according to the following equation:

RSC (%) = [(A_control_ − A_sample_)/A_control_] × 100 (2)

where A_control_ and A_sample_ are the absorbance values of the control and the sample, respectively. The IC50 value showing the concentration that caused 50% scavenging of the DPPH^•^ and ABTS^•+^ radical was calculated from the graph, plotted as RSC percentage against the extract concentration. All experiments were carried out in triplicate and at least on three separate occasions.

### 2.5. ABTS^•+^ Radical Scavenging Assay

The 2,2′-azino-bis(3-ethylbenzthiazoline-6-sulfonic acid) (ABTS^•+^) radical scavenging assay was carried out as described previously [43]. In brief, the ABTS^•+^ radical was generated by mixing 2 mM ABTS with 30 μM H_2_O_2_ and 6 μM horseradish peroxidase (HRP) enzyme in 1 mL of distilled water. The reagents were mixed and incubated at room temperature in the dark for 45 min. Each macroalgae extract was dissolved in double distilled water to make stock solutions (300 mg/mL). These stocks were used for achieving different extract concentrations by making serial dilutions. Then, 10 μL of different extract concentrations in aqueous solution were added in the reaction mixture, and the absorbance at 730 nm was read. In each experiment, the tested sample in distilled water containing ABTS and H_2_O_2_ was used as a negative control. These negative controls were used to avoid the possible interference of the extract’s absorbance by itself, with the absorbance measured by the assay. The absorbance of these negative controls was subtracted by the absorbance of the corresponding samples. The ABTS^•+^ radical solution with 10 μL H_2_O was used as control. Ascorbic acid was used as a positive control for the antioxidant activity. The percentage of RSC of the tested extracts was calculated as described above for the DPPH assay. At least three independent experiments were performed for each tested compound.

### 2.6. Hydroxyl Radical Scavenging Assay

Hydroxyl radical (^•^OH) scavenging activity was determined as described previously [44]. In brief, 75 μL of extract dissolved in distilled water at different concentrations was added to 450 μL sodium phosphate buffer (0.2 M, pH 7.4), 150 μL 2-deoxyribose (10 mM), 150 μL FeSO_4_-EDTA (10 mM), 525 μL H_2_O, and 150 μL H_2_O_2_ (10 mM), and the samples were incubated at 37 °C for 4 h. After incubation, 750 μL trichloroacetic acid (TCA) (2.8%) and 750 μL 2-thiobarbituric acid (1%) were added, and the samples were incubated at 95 °C for 10 min. The samples were cooled on ice for 5 min and centrifuged at 3000 rpm for 10 min at 25 °C. The absorbance was measured at 520 nm. In each experiment, the samples without H_2_O_2_ were used as negative controls. These negative controls were used to avoid the possible interference of the extract’s absorbance by itself with the absorbance measured by the assay. The absorbance of these negative controls was subtracted by the absorbance of the corresponding samples. The samples without extract were used as controls. Ascorbic acid was used as a positive control for the antioxidant activity. The OH^•^ radical scavenging activity was calculated according to the following equation:

^•^OH radical scavenging activity (%) = [(Abs_control_ – Abs_sample_)/Abs_control_] × 100 (3)

where Abs_control_and Abs_sample_ are the absorbance values of the control and the tested sample, respectively. At least three independent experiments were performed for each tested compound.

### 2.7. Superoxide Radical Scavenging Assay

The superoxide anion radical (O_2_^•−^)-scavenging activity of the extracts was evaluated as described previously [45] with minor modifications. Specifically, in this method, O_2_^•−^ radicals are produced by the phenazine methosulfate and reduced nicotinamide adenine dinucleotide (PMS-NADH) system by NADH oxidation, and then they reduce nitroblue tetrazolium (NBT) to formazan, which is measured spectrophotometrically at 560 nm. Antioxidants may scavenge O_2_^•−^, consequently reducing absorbance. For this assay, the macroalgae extracts were dissolved at different concentrations in Tris-HCl of 16 mM (pH 8.0), which was the buffer. More specifically, 125 μL of NBT_2_^+^ (300 μΜ), 125 μL of NADH (468 μΜ), and 10 μL of extracts (diluted in the buffer) were added into 615 μL of Tris-HCl (16 mM; pH 8.0). The reaction was initiated by the addition of 125 μL of PMS (60 μΜ) to the mixture. The samples were incubated for 5 min in the dark, and the absorbance was monitored at 560 nm on a Perkin Elmer Lambda 25 UV/VIS spectrophotometer (Waltham, MA, USA). In each measurement, a blank containing 750 μL of Tri-HCl buffer, 125 μL of NBT, and 125 μL of NADH, and a control containing 625 μL of Tri-HCl buffer, 125 μL of NBT, 125 μL of NADH, and 125 μL of PMS were used. Moreover, in each experiment, negative controls were used containing 740 μL of Tri-HCl buffer, 125 μL of NBT, 125 μL of NADH, and 10 μL of extract diluted in buffer. These negative controls were used to avoid the possible interference of the extract’s absorbance by itself with the absorbance measured by the assay. The absorbance of these negative controls was subtracted by the absorbance of the corresponding samples. The RSC and the IC_50_ values for O_2_^•−^ were evaluated as mentioned above for the DPPH^•^ radical. At least three independent experiments were performed for each tested compound.

### 2.8. RP Assay

Reducing power was determined spectrophotometrically as described previously [44] with minor modifications. In this assay, the macroalgae extracts were dissolved in phosphate buffer (0.2 M, pH 6.6) at different concentrations. Two hundred and fifty microliters of the extract solution was added to 250 μL of potassium ferricyanide (1% *w*/*v* in dH_2_O) and incubated at 50 °C for 20 min. After incubation, the samples were cooled on ice for 5 min. Then, 250 μL of TCA (10 *w*/*v*) was added, and the samples were centrifuged (1700 g, 10 min, 25 °C). Subsequently, 250 μL of distilled H_2_O and 50 μL of ferric chloride (0.1% *w*/*v*) were added to the supernatant, and the samples were incubated at room temperature (RT) for 10 min. The absorbance was monitored at 700 nm on a Perkin Elmer Lambda 25 UV/VIS spectrophotometer (Waltham, MA, USA). In each measurement, a blank containing 500 μL of phosphate buffer, 250 μL of TCA, 250 μL of dH_2_O, and 50 μL of ferric chloride, and a control containing 250 μL of buffer, 250 μL of potassium ferricyanide, 250 μL of TCA, 250 μL of dH_2_O, and 50 μL of ferric chloride were used. Moreover, in each experiment, negative controls were used containing 250 μL of buffer, 250 μL of TCA, 250 μL of dH_2_O, and 50 μL of ferric chloride and 250 μL of extract diluted in buffer. These negative controls were used to avoid the possible interference of the extract’s absorbance by itself with the absorbance measured by the assay. Ascorbic acid was used as a positive control for the RP activity. The absorbance of these negative controls was subtracted by the absorbance of the corresponding samples. The RP_0.5AU_ value showing that the extract concentration caused an absorbance of 0.5 at 700 nm was calculated from the graph plotting absorbance against extract concentration. At least three independent experiments were performed for each tested compound.

### 2.9. ROS-Induced DNA Plasmid Strand Cleavage Assay

The ROS-induced DNA plasmid strand cleavage assay was performed as described previously [18]. At least three independent experiments were performed for each tested compound.

### 2.10. Evaluation of Relative Antioxidant Capacity Index (RACI)

The assessment of the order of the antioxidant potency of macroalgae extracts, taking into account their activity in all antioxidant assays, was based on the evaluation of the RACI for each extract, as described previously [46]. Since RACI estimation was based on IC_50_ values and RP_0.5AU_ values, the lower the RACI value was, the higher the antioxidant capacity was.

### 2.11. Cell Culture Conditions

The human liver HepG2 cancer cell line was obtained from Dr. Anna-Maria Psarra (University of Thessaly, Larissa, Greece). The cells were cultured in normal Dulbecco’s modified Eagle’s medium (DMEM; Gibco, Horsham and Loughborough, UK) containing 10% (*v*/*v*) fetal bovine serum, 2 mM L-glutamine (Gibco, Horsham and Loughborough, UK), 100 units/mL of penicillin, and 100 units/mL of streptomycin (Gibco, Horsham and Loughborough, UK) in plastic disposable tissue culture flasks at 37 °C in 5% CO_2_.

### 2.12. XTT Assay for Inhibition of Cell Proliferation 

The inhibition of cell proliferation was assessed using the XTT assay kit (Roche, Germany), as described previously [43]. Briefly, 1 × 10^4^ cells were subcultured into a 96-well plate in DMEM medium. After 24 h incubation, the cells were treated with different concentrations of macroalgae extracts in serum-free DMEM medium for 24 h. Then 50 μL of XTT test solution, which was prepared by mixing 50 μL of XTT-labeling reagent with 1 μL of electron coupling reagent, was then added to each well. After 4 h of incubation, absorbance was measured at 450 nm and also at 690 nm as a reference wavelength on a Perkin Elmer EnSpire Model 2300 Multilabel microplate reader (Waltham, MA, USA). Cells cultured in DMEM serum-free medium were used as a negative control. Additionally, the absorbance of each extract concentration alone in DMEM serum-free medium and XTT test solution was tested at 450 nm. The absorbance values shown by the extracts alone were subtracted from those derived from cancer cell treatment with extracts. Data were calculated as percentage of inhibition by the following formula:

Inhibition (%) = [(O.D._control_ − O.D._sample_)/O.D._control_] × 100 (4)

where O.D._control_ and O.D._sample_ indicated the optical density of the negative control and the tested substances, respectively. The concentration of macroalgae extracts causing 50% cellular proliferation inhibition (IC_50_) of cancer cells was calculated thereafter from the graph plotted percentage inhibition against extract concentration. All experiments were carried out at least on three separate occasions in triplicate.

### 2.13. Statistical Analysis

All results were expressed as mean ± SD. For statistical analysis, one-way ANOVA was applied followed by Dunnett’s test for multiple pair-wise comparisons. Dose–response relationships were examined by Spearman’s correlation analysis. 

Spearman’s correlation was also used to determine the correlation between the values of different bioactivity assays and TPC values. Correlation coefficients whose magnitudes were less than 0.49, from 0.5 to 0.69, and from 0.7 to 1.0 were considered as having low, medium, and high correlations, respectively. 

Differences were considered significant at *p* < 0.05. All statistical analyses were performed with the SPSS software (version 14.0; SPSS).

In order to identify clusters of closely related macroalgae species, in terms of their overall bioactivities, dendrograms and principal component analysis (PCA) were performed. Clustering was based on seven measures (i.e., DPPH^•^, ABTS^•+^, OH^•^, O_2_^•−^, RP, DNA plasmid breakage, and XTT assay for HepG2 cells). Dendrograms were generated using the Euclidian Distance metric and the WPGMA algorithm, with the Scipy python package [47]. PCA was conducted with the scikit-learn package [48] for two components using default parameters. Dendrograms and PCA plots were generated with the plotly Python package (Plotly Technologies Inc., Collaborative data science, Montréal, QC, Canada, 2015. https://plot.ly; accessed on 30 January 2023), for raw as well as normalized data, based on the Z-score transformation. All the above clustering analyses were initially conducted for all fourteen macroalgae. However, after one outlier sample was detected (i.e., *C. fragile*), all the above analyses were also repeated for the remaining thirteen samples.

## 3. Results and Discussion

### 3.1. Extraction Yield and Assessment of Polyphenolic Content

The extraction yields ranged from 18.0% (*U. rigida*) to 46.7% (*Codium fragile*) (Table 2). The average of extraction yield was 24.9 ± 7.8%. Only two species exhibited significant deviation from the mean value, that is, the green macroalgae *C. fragile* (46.7%) and the brown macroalgae *C. amentacea* (34.1%). Moreover, on average, we did not observe significant differences in the yields between red, brown, and green macroalgae. Since the same extraction method was used for all macroalgae, differences in extraction yield may be due to differences in the macroalgae’s chemical composition [16,49]. Although the comparison of extraction yield between different studies is difficult due to various methods and solvents used, our yield values were comparable with those of other studies. For example, *G. gracilis*’ yield of extraction using hot water was 24.63% [50], being close to our yield of 25.4% after 80% *v*/*v* methanol extraction. In addition, our *C. amentacea*’s extraction yield of 34.1% was similar to the yield of 31% of macroalgae after 50% *v*/*v* ethanol extraction [51]. Our *C. barbata*’s yield of 22.4% was also close to the yield of 24.31% of extract obtained in 70% *v*/*v* acetone [52]. However, intriguingly, 100% *v*/*v* methanol was used for extraction, *C. barbata*’s yield was too low (3.8%) [52]. 

Since algal polyphenols are known for their antioxidant and/or anticancer activities [7], macroalgae extracts’ TPC values were assessed. The results showed that the extracts had low TPC values and ranged about 23-fold, from 0.55 to 12.53 mg GAE/g dw of extract (Table 2). The *G. teedei* extract exhibited the highest TPC value (12.53 mg GAE/gr dw) followed by *C. barbata* (5.76 mg GAE/gr dw) and U. rigida (4.15 mg GAE/gr dw) (Table 2). Two extracts had TPC values below 1 mg GAE/g dw of extract, six extracts from 2 to 3 mg GAE/g dw, three extracts from 3 to 4 mg GAE/g dw, and three extracts above 4 mg GAE/g dw (Table 2). 

The TPC values of several macroalgae were similar to those found in other studies. For example, Francavilla et al. [53] reported that the TPC of a *G. gracilis* methanolic extract was 2.3 mg GAE/dw, that is, it was very close to our methanolic extract (3.01 mg GAE/dw). Interestingly, Francavilla et al. [53] collected *G. gracilis* from the Mediterranean Sea (Southern Adriatic Sea, Lesina, Italy) like us. However, in the aforementioned study, TPC values varied significantly between different solvents used for extraction. For example, their ethyl acetate extract had a TPC of ~65 mg GAE/dw [53]. Moreover, Sapatinha et al. [50] demonstrated that *G. gracilis* extracts had TPCs from 28.2 to 50.73 mg GAE/dw, but they used different solvents than we did.

Furthermore, the TPC of methanolic *P. pavonica* extract was 0.96 GAE/dw [54], a value close to our result (2.77 mg GAE/gr dw). However, *P. pavonica* was also reported to contain a higher TPC (27 mg GAE/gr dw) than our value, but acetone was used for the extraction instead of methanol [55]. 

In addition, S. vulgare extract was demonstrated to have a TPC of 6.60 mg GAE/g dw, being about 3-fold higher than that of our extract, but dichloromethane along with methanol was used for extraction [56]. 

Additionally, De La Fuente et al. [51] reported a TPC of 20.3 mg GAE/g dw for *C. amentacea* methanolic extract, while our extract had a much lower value (2.54 mg GAE/g dw). Moreover, methanolic *C. compressa* extract contained 0.161 mg GAE/g dw [27], which was much lower than that of our extract (2.93 mg GAE/g dw). Interestingly, Guner [27] also collected *C. compressa* from the Aegean Sea (Coast of Urla, Izmir, Turkey), but from a different region than ours. In addition, another study showed that the TPC of *C. compressa* extracts varied according to season from 48.2 to 83.4 mg GAE/g [57], that is, the values were higher than our finding (2.93 mg GAE/g dw). In that case, although the same solvent as ours was used, microwave extraction was carried out [57], a method completely different than that used in our study. In general, apart from the extraction method [50,53], several other factors may affect macroalgae’s TPC values, such as seasonality [57], local environmental conditions (e.g., salinity, nutrient availability, UV irradiation, and light), and geographical location [50,58,59,60]. 

Neto et al. [61] showed U. rigida extracts’ TPCs to range from 1.6 to 5.3 mg GAE/g dw, depending on the extraction method. These values were close to that of U. rigida in our study (4.15 mg GAE/g dw). Additionally, Megzhani et al. [62] reported U. rigida extract’s TPCs (3.29 mg GAE/g dw) to be similar to this study, although their ethanolic extract exhibited a higher value (8.09 mg GAE/g dw). In addition, Farasat et al. [42] used exactly the same extraction method as us for *U. intestinalis* and found a similar TPC value (i.e., 1.98 vs. 2.11 mg GAE/g dw). A *U. intestinalis* ethanolic extract was demonstrated to contain a TPC of 1.15 mg GAE/g dw [63], similar to our value. However, the same researchers showed a higher TPC (11.27 mg GAE/g dw) of *U. intestinalis* than this study when samples were treated by ultrasonication [63]. In another study, the accelerated solvent method was used for *U. intestinalis* extraction, and the TPC value was 5 mg GAE/g dw [64]. The green macroalgae *C. fragile* was also shown previously to have a TPC of 0.99 mg GAE/g dw [65], similar to this study (0.55 mg GAE/g dw). 

For identifying individual compounds accounting for the observed bioactivities of the macroalgae extracts, the presence of ten polyphenols or simple phenols was investigated with HPLC-DAD analysis. These phenols were caftaric acid, caffeic acid, epigallocatechine gallate, p-coumaric acid, chicoric acid, trans-ferulic acid, quercetin, sinapic acid, rutin, and trans-cinnamic acid. These polyphenols were examined, since all of them have been found in macroalgae species, including those tested in the present study [66,67,68,69,70]. The results showed that none of the macroalgae extracts’ chromatograms showed detectable peak areas of the standard phenols (Supplementary Figure S1). Thus, the polyphenols or phenols under investigation were not contained in any of the tested macroalgae extracts.

In another study, the results of HPLC-MS/MS carried out for identifying compounds of *C. amentacea* extract showed as main components meroditerpene-like structures [51]. Moreover, Caf et al. [54], in agreement with our study, did not identify rutin using HPLC in *P. pavonica* collected from the Eastern Mediterranean Sea (Lara coast, Antalya, Turkey) like our sample. However, unlike our study, they detected quercetin, but its amount was low (0.013 μg/g dw extract) [54]. Other phenols identified by Caf et al. [54] in *P. pavonica* were myrisetin (0.034 μg/g dw), morin (0.011 μg/g dw), naringenin (0.065 μg/g dw), and resveratrol (0.11 μg/g dw), while kaempferol and naringin were not found. In another study on *P. pavonica* collected from the Mediterranean Sea (coast of Ciovo Island, Croatia), unlike our extract, p-coumaric and trans-ferulic acids were identified in extracts derived from different methods, but at low amounts ranging from 0.02 to 0.88 mg/L and from 0.07 to 1.22 mg/L extract, respectively [71]. These extracts were obtained using a method (ultrasound-assisted extraction in ethanol or water) [71] different to what was used in our study. Other polyphenols identified in this study were protocatechuic acid (from 1.05 to 1.70 mg/L extract) and p-hydroxybenzoic acid (from 0.51 to 0.76 mg/L extract) [71]. Furthermore, an interesting study used quantitative ^1^H NMR (qNMR), a very sensitive method compared to HPLC-DAD, for polyphenols’ identification in *U. intestinalis* and, similar to our results, did not find chicoric acid but identified small amounts of sinapic acid, ferulic acid, p-coumaric acid, quercetin, caffeic acid, gallic acid, luteolin, apigenin, and diosmetin [64]. They also concluded that polyphenols’ identification from marine macroalgae presents many difficulties due to there being complex samples and polyphenols’ presence at low concentrations [64]. The differences in polyphenols’ determination between our study and other studies may be attributed to different factors such as geographical location and season of collection, as well as extraction and chemical analysis methods [50,51,56].

### 3.2. Free Radical Scavenging Activity 

Since oxidative stress has been shown to be a causative factor for cancer [72], macroalgae extracts’ scavenging abilities against four different free radicals (i.e., DPPH, ABTS^•+^, ^•^OH, and O_2_^•*−*^) were determined. The IC_50_ values of all assays are shown in Table 2. The lower the IC_50_ value, the higher the antioxidant activity.

In DPPH^.^ and ABTS^•+^, as expected, ascorbic acid as a pure compound had lower IC_50_ values than the extracts. Interestingly, in the ^•^OH assay, *G. pistillata*, *G. teedei*, and *C. amentacea* exhibited better scavenging activity than ascorbic acid. In the O_2_^•−^ assay, ascorbic acid could not be tested because it can reduce NBT [73].

In DPPH assay, the macroalgae species’ IC_50_ values ranged from 0.31 to 79.00 mg/mL (Table 2). The three most potent macroalgae species against DPPH^•^ scavenging were *G. teedei* (IC_50_: 0.31 mg/mL), *C. barbata* (IC_50_: 1.40 mg/mL), and *G. pistillata* (IC_50_: 2.10 mg/mL) (Table 2). Our results were partly similar to those of other studies, but different findings from ours have also been reported. For example, Francavilla et al. [53] demonstrated that the *G. gracilis*’ IC_50_ value against DPPH^.^ varied according to the solvent used for the extraction and season of macroalgae collection. When methanol was used for extraction, IC_50_ values ranged from 2.94 to 9.72 mg/mL [53], similar to our study (13.5 mg/mL). However, Sapatinha et al. [50] and Zubia et al. [74] reported *G. gracilis*’ IC_50_ values of ~80 and 42.27 mg/mL, respectively, in the DPPH^.^ assay, which were significantly higher than our IC_50_ values. Moreover, *C. amentacea* extracts isolated with 50% *v/v* ethanol and dimethyl sulfoxide (DMSO) exhibited in the DPPH assay IC_50_ values of 205.1 and 0.34 μg/mL, respectively [51], which were much lower than in this study (2.5 mg/mL). Kosanic et al. [75] reported an DPPH^.^ IC_50_ value of 409.81 μg/mL of the *C. amentacea* extract, which was also lower than this study, but they used a different solvent and method (i.e., acetone in a Soxhlet extractor) for extract isolation than us. Additionally, *C. barbata*’s IC_50_ values in DPPH^.^ assay varied according to solvent used for extraction from 0.088 to 0.564 mg/mL [52,75], being lower than in this study (1.4 mg/mL). Guner et al. [27] demonstrated DPPH^.^ IC_50_ values of 15.94, 5.00, and 7.46 mg/mL of *C. compressa* extracts isolated using methanol, hexane, and chloroform, respectively, which were higher than our IC_50_ (2.9 mg/mL). Interestingly, Guner et al. [27] collected *C. compressa* from the Aegean Sea like us. However, Mhadhebi et al. [76] documented a DPPH^.^ IC_50_ value of 0.012 mg/mL for *C. compressa* collected from the Tunisian coastline in the Mediterranean Sea. Kosanic et al. [75] also reported *C. compressa*’s DPPH^.^ IC_50_ value of 812.22 μg/mL. De La Fuente et al. [56] attributed *C. compressa*’s antioxidant activity, at least in part, to a sulphated polysaccharide extract with a DPPH^.^ IC_50_ of 142.5 μg/mL. *P. pavonica* isolated in 95% *v/v* ethanol was shown in the DPPH^.^ assay to possess an IC_50_ of 5.59 μg/mL [77], a value much lower than our result (6.5 mg/mL). Chouh et al. [78] demonstrated a DPPH^.^ IC_50_ value of 97.41 μg/mL of S. vulgare extract, which was also lower than our value (8.2 mg/mL). However, they used 70% *v/v* acetone for extraction [50] instead of 80% *v/v* methanol, which we used. Interestingly, De La Fuente et al. [56] showed an extract of sulphated polysaccharides from *S. vulgare* from the Mediterranean Sea to exhibit an IC_50_ of 695.5 μg/mL. Mezghani et al. [62] reported DPPH^.^ IC_50_ values of *U. rigida* ranging from 204.08 to 500 μg/mL, depending on the extraction solvent, while our value was 5.5 mg/mL. A methanolic extract of *U. intestinalis* was demonstrated to have a DPPH^.^ IC_50_ value of 1.88 mg/mL [42], while our value was 10 mg/mL.

Macroalgae extracts’ IC_50_ values against ABTS^•+^ scavenging were from 0.02 to 15.00 mg/mL (Table 2). Among tested algae extracts, *G. teedei* (IC_50_: 0.024 mg/mL), *G. pistillata* (IC_50_: 0.16 mg/mL), and *P. pavonica* (IC_50_: 0.38 mg/mL) exhibited the lowest IC_50_ values. Trifan et al. [52] documented *C. barbata* extracts’ ABTS^•+^ IC_50_ values to range from 13.9 to 22.1 μg/mL, while our value was higher (0.43 mg/mL). *G. gracilis*’ IC_50_ values in the ABTS assay ranged from ~15 to 30 mg/mL depending on the solvent and method used for extraction [50], while our value was lower (1.45 mg/mL). Chouh et al. [78] demonstrated S. vulgare extract’s ABTS IC_50_ value of 72.9 μg/mL, which was lower than ours (1.4 mg/mL). 

Moreover, all macroalgae extracts scavenged the ^•^OH radical with IC_50_ values ranging from 0.10 to 10.00 mg/mL (Table 2). In this assay, the macroalgae species exhibiting the highest activity were *G. teedei* (IC_50_: 0.10 mg/mL), *G. pistillata* (IC_50_: 0.14 mg/mL), and *C. amentacea* (IC_50_: 0.16 mg/mL). De La Fuente et al. [51] documented, in an ^•^OH assay, IC_50_ values of 0.29 and 0.45 mg/mL for *C. amentacea* extracts isolated with 50% *v/v* ethanol and DMSO, respectively, which were close to our value (0.16 mg/mL).

In addition, in the O_2_^•−^ radical scavenging assay, macroalgae extracts’ IC_50_ values ranged from 0.05 to 6.40 mg/mL (Table 2). In this assay, the three most potent extracts were *G. teedei* (IC_50_: 0.05 mg/mL), *G. pistillata* (IC_50_: 0.07 mg/mL), and G. bursa pastoris (IC_50_: 0.14 mg/mL) (Table 2). Unlike all the other scavenging assays, two macroalgae species, that is, *U. rigida* and *U. intestinalis*, could not achieve IC_50_ values at the tested concentrations. Actually, it was not possible to determine IC_50_ values for these two species. The reason was that at concentrations higher than 0.2 mg/mL, their extracts formed a precipitate, probably due to a reaction of one of their compounds with the reaction mixture, which impeded absorbance measurement. Thus, at 0.2 mg/mL, *U. rigida* scavenged O_2_^•−^ by 32%, while the value for *U. intestinalis* was 43.20%. Chouh et al. [78] demonstrated, in an O_2_^•−^ radical assay, IC_50_ value of >800 μg/mL of S. vulgare extract, while our value was 600 μg/mL. Kosanic et al. [75] reported, in an O_2_^•−^ radical assay, for *C. barbata*, *C. amentacea*, and *C. compressa* extracts isolated with acetone in a Soxhlet extractor, IC_50_ values of 675.93, 521.45, and 976.62 μg/mL, respectively, while our values were 1.2, 1.4, and 1.1 mg/mL, respectively. 

It was remarkable that all extracts were less potent in DPPH^.^ assays compared to the other three free radical scavenging assays. The solvent of the DPPH^.^ assay is methanol, while the solvent of the other three assays is water. Thus, lipophilic compounds are mainly active in the DPPH^.^ assay, while hydrophilic compounds are more potent in ABTS^•+^, ^•^OH, and O_2_^•−^ assays. Consequently, it may be concluded that the antioxidant compounds of the tested macroalgae extracts are mainly hydrophilic. Both DPPH^.^ and ABTS^•+^ assays are based on synthetic radicals, but they consist of the most frequent methods used to determine the antioxidant activity of a compound [79]. On the contrary, ^•^OH and O_2_^•−^ radicals are formed naturally in the human organism [80]. The overproduction of O_2_^•−^ within cells results in reactions with biological macromolecules, causing damage to cellular components and dysfunction of cell metabolism [72]. Moreover, intracellular superoxide dismutase (SOD) can catalyze O_2_^•−^ to hydrogen peroxide (H_2_O_2_) reacting through the Fenton reaction with Fe^2+^, leading to formation of ^•^OH that may cause DNA damage and cancer [72]. Thus, the identification of compounds being able to scavenge both ^•^OH and O_2_^•−^ radicals is of great importance for cancer prevention. Finally, it should be noted that in all free radical scavenging assays, there was a great variation in potency among the tested macroalgae extracts. However, it was obvious from the IC_50_ values in all assays that the two *Gigartina* species, *G. teedei* and *G. pistillata*, had higher free radical scavenging activity than the other extracts. 

### 3.3. RP Activity

**Figure 2:**
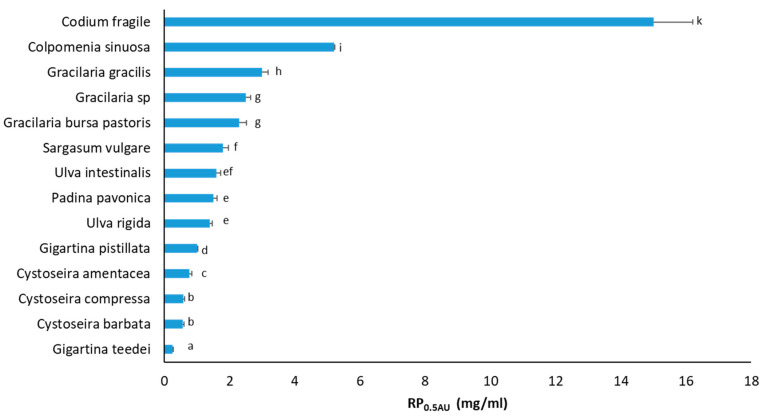
RP activity of macroalgae extracts. The RP_0.5AU_ values are presented as the mean ± SD from at least three independent experiments. All RP_0.5AU_ values are statistically significantly (*p* < 0.05) different compared to controls. Values having different letters are statistically different between them (*p* < 0.05).

Macroalgae extracts’ RP values were determined, since the ability of bioactive compounds to act as electron donors is considered as an indication of their capacity to neutralize free radicals [79]. In the RP assay, tested extracts’ RP_0.5AU_ values ranged from 0.24 to 15 mg/mL (Figure 2). It should be noted that similar to IC_50_ values, the lower the RP_0.5AU_ value, the higher the RP activity. The three species demonstrating the highest reducing activity were *G. teedei* (RP_0.5AU_: 0.24 mg/mL), *C. barbata* (RP_0.5AU_: 0.56 mg/mL), and *C. compressa* (RP_0.5AU_: 0.58 mg/mL) (Figure 2). Since ascorbic acid is a pure compound, it exhibited an RP_0.5AU_ of 3.4 μg/mL (data not shown), being much lower compared to extracts. 

Like free radical scavenging assays, macroalgae extracts’ RP exhibited great variation. For example, *G. teedei*, the most potent extract, exhibited a 62.5 times greater reducing activity than *C. fragile*, the least potent extract. In general, the two *Gigartina* species along with the three *Cystoseira* species had RP_0.5AU_ values below or equal to 1 mg/mL, the two *Ulva* species together with *P. pavonica* and *S. vulgare* had RP_0.5AU_ from 1 to 2 mg/mL, while the three *Gracilaria* species, *C. sinuosa* and *C. fragile*, exhibited RP_0.5AU_ values higher than 2 mg/mL. The fact that the *G. teedei* extract, like in all free radical scavenging assays, was the most potent in the RP assay confirmed the association between reducing activity and free radical neutralization. Therefore, the results suggested that the *G. teedei* extract’s antioxidant compounds may also be effective electron donors. 

In other studies, De La Fuente et al. [51] documented for *C. amentacea* extracts isolated with DMSO or 50% *v/v* ethanol RP_0.5AU_ values of 0.11 and 0.64 mg/mL, respectively. The latter value was comparable to our value (0.77 mg/mL). Chouh et al. [78] demonstrated an RP_0.5AU_ value of >200 μg/mL of S. vulgare extract, while our value was 1.8 mg/mL. 

### 3.4. Protection from ROS-Induced DNA Damage 

**Figure 3:**
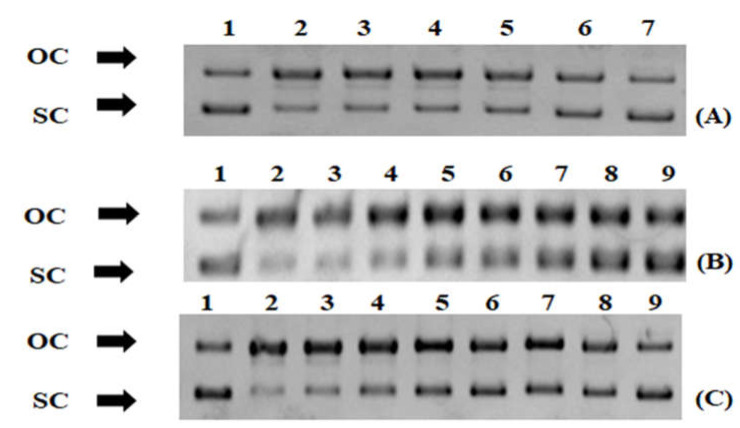
Representative photo of plasmid DNA gel electrophoresis showing protective activity of macroalgae extracts against ROO^•^ radical: (**A**) *G. teedei*. Lane 1, plasmid DNA alone; lane 2, plasmid DNA with addition of ROO^•^ radical; lanes 3–6, plasmid DNA with addition of ROO^•^ radical along with different concentrations of extract (0.008, 0.016, 0.032, and 0.064 mg/mL); lane 7, plasmid DNA with addition of the extract alone at the maximum tested concentration; (**B**) *U. rigida*, (**C**) *G. bursa pastoris.* Lane 1, plasmid DNA alone; lane 2, plasmid DNA with addition of ROO^•^ radical alone; lanes 3–8 plasmid DNA with addition of ROO^•^ radical along with different concentrations of extract (*U. rigida*: 0.063, 0.125, 0.250, 0.500, 1.0, and 2.0 mg/mL); (*G. bursa pastoris*: 0.125, 0.250, 0.500, 1.0, 2.0, and 4.0 mg/mL); lane 9, plasmid DNA exposed with addition of the extract alone at the maximum tested concentration; OC: open circular; SC: supercoiled.

**Figure 4:**
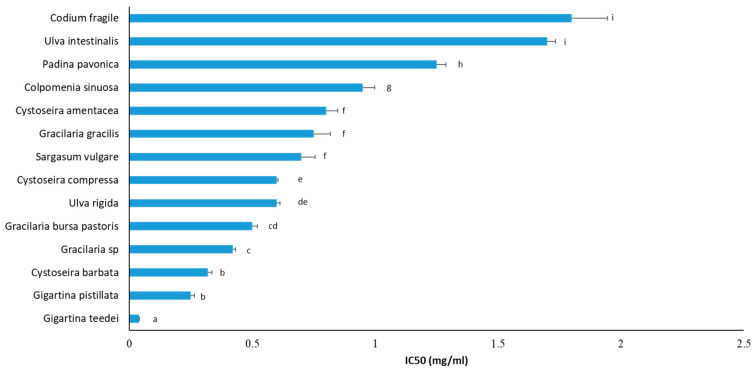
Protection from ROS-induced DNA damage of macroalgae extracts. The IC_50_ values are presented as the mean ± SD from at least three independent experiments. All IC_50_ values are statistically significant (*p* < 0.05) compared to controls. Values having different letters are statistically different between them (*p* < 0.05).

The evidence of macroalgae extracts’ antioxidant potential was further supported by their ability to protect from ROO^•^-induced DNA damage (Figure 3 and Figure 4). The IC_50_ values in this assay ranged from 0.038 to 1.8 mg/mL (Figure 4). The most potent extract, such as free radical scavenging assays, was *G. teedei* (IC_50_: 0.038 mg/mL) followed by *G. pistillata* (IC_50_: 0.25 mg/mL) and *C. barbata* (IC_50_: 0.32 mg/mL) (Figure 4). Interestingly, IC_50_ values of the DNA plasmid breakage assay were on average lower than IC_50_ values of all free radical scavenging assays and RP_0.5AU_ values. 

The ROO^•^ radicals used for DNA damage are usually produced in cells by the reaction of oxygen with radicals containing carbon atoms [81]. Then, after their entrance to the nucleus, they may cause DNA damage and diseases such as cancer [81]. To the best of our knowledge, this is the first study to demonstrate for the most of the tested macroalgae species’ extracts protection from ROS-induced DNA damage. Only for *C. barbata* was a sulphated polysaccharide extract reported to inhibit DNA damage caused by (^•^OH) at a concentration of 0.125 mg/mL [82], which was close to our IC_50_ value (0.32 mg/mL). Moreover, the *U. rigida* ethanolic extract was shown to protect bone marrow cells from genotoxicity [83]. Since it is well established that DNA damage is a crucial factor for cancer manifestation and progression [84], the identification of compounds protecting from ROS-induced DNA damage is of great importance.

### 3.5. Estimation of RACI Values

**Figure 5:**
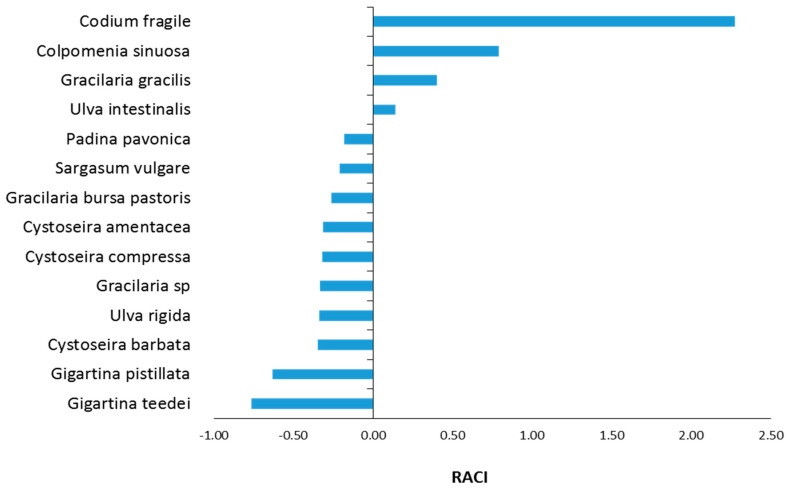
Potency order of macroalgae extracts’ antioxidant activity based on RACI values.

Since for the assessment of macroalgae extracts’ antioxidant capacity six different antioxidant assays (i.e., DPPH, ABTS^•+^, ^•^OH, O_2_^•−^, RP, and DNA plasmid strand cleavage) were used and in each assay the extracts’ potency order was different, it was difficult to find out which extract was the most potent. Thus, for estimating the macroalgae extracts’ potency order by combining the values of all the above assays, the RACI was estimated for each macroalgae species (Figure 5). The RACI estimation showed that its values ranged from −0.77 to 2.28. As mentioned, the lower the RACI value, the higher the antioxidant capacity. Thus, the most potent antioxidant extract was *G. teedei* (−0.77) followed by *G. pistillata* (−0.63), Cystoseira barbata (−0.35), and *U. rigida* (−0.34) (Figure 5).

Moreover, these four species exhibited higher TPCs (Table 2), and so their polyphenolic amounts may account for their high antioxidant activity. Other studies have also shown that macroalgae’s antioxidant activity is attributed to their polyphenols [7,51,85]. For example, *G. gracilis* [53], *C. amentacea*, *C. barbata*, and *C. compressa* [75] extracts’ polyphenolic contents accounted for their antioxidant activity. Specifically, phlorotannins (e.g., phloroglucinol) and flavonoids have been demonstrated to be strong antioxidants in several macroalgae species such as *S. vulgare*, *P. pavonica*, and *C. barbata* [7,52,71,78]. Additionally, Trifan et al. [52] identified 18 phlorotannins in *C. barbata* extracts exhibiting antioxidant activity. These phlorotannins were mainly fucophlorethol and eckol derivatives, containing between three and seven phloroglucinol units [52]. Moreover, *P. pavonica* extracts contained polyphenols such as quercetin, resveratrol, trans-ferulic acid, and p-hydroxybenzoic acid, known for their antioxidant activity [86]. Chouh et al. [78] identified in *S. vulgare* 21 phlorotannins such as dibenzodioxine1,3,6,8-tetraol, fuhalol, pentaphlorethol, fucopentaphlorethol, and dihydroxypentafuhalol with antioxidant properties. *G. pistillata* has also been reported to contain antioxidant polyphenols such as (–)–epicatechin, protocatechuic acid, oleuropein, p-aminobenzoic acid, and tyrosol [87]. *U. intestinalis* extracts have been reported to contain antioxidant polyphenols such as sinapic acid, ferulic acid, p-coumaric acid, quercetin, caffeic acid, gallic acid, luteolin, apigenin, and diosmetin [64,86]. Further evidence of our results for polyphenols’ roles in the tested macroalgae’s antioxidant potency was that *C. fragile* and *C. sinuosa* extracts exhibiting the least antioxidant activity had also the lowest TPC values.

Apart from polyphenols, other algal compounds have also been shown to possess antioxidant activity. Specifically, the most important macroalgae’s metabolites accounting for their antioxidant activity are phenols (e.g., phlorotannins, flavonoids, phenolic acids, and bromophenols), polysaccharides (e.g., carrageenans, sulfated polysaccharides, agar, fucoidan), fatty acids, phytosterols, proteins (e.g., phycobiliproteins), terpenoids (e.g., carotenoids, zeaxanthin), pigments (e.g., chlorophylls), and iodine [87]. For instance, sulfated polysaccharides (e.g., fucoidan and alginate) from *C. sinuosa*, *C. barbata*, and *U. rigida* exhibited antioxidant activity [82,88,89,90,91,92]. Red macroalgae such as *Gigartina* and *Gracilaria* species are also rich in carrageenans and sulfated polysaccharides, demonstrating antioxidant properties [93]. Specifically, G*. pistillata* has been found to contain carrageenans such as hybrid kappa-iota and xi-lambda carrageenans [94]. Moreover, sulfated polysaccharides with free radical scavenging activity have been identified in *G. gracilis* [95]. These polysaccharides consisted mainly of galactose, ribose, arabinose, and glucose [95]. In addition, the antioxidant activity of *C. compressa*’s extracts was attributed to polysaccharides such as fucoidan and monosaccharides such as fucose, galactose, and mannose [96]. In *C. fragile*, sulfated polysaccharides, which are mainly linear homopolymers comprising ß-1.4-linked D-mannose residues, were shown to possess antioxidant activity by promoting survival while decreasing ROS, cell mortality, and lipid peroxidation in a zebrafish experimental model [97]. Furthermore, *C. compressa*’s extracts exhibiting antioxidant activity were reported to contain a series of fatty acids such as oleic acid, palmitoleic acid (C16:1n-7), palmitic acid (C16:0), and ω-3 eicosapentaenoic acid (EPA) [57]. *Cystoseira amentacea* extracts’ antioxidant activity has been attributed to terpenoids such as meroditerpenes and linear diterpenes [51]. Moreover, *P. pavonica*, *C. barbata*, and other brown macroalgae contain phytosterols such as fucosterol, exhibiting antioxidant activity [52,55]. In addition, red macroalgae (e.g., *G. gracilis*) have been reported to contain phycobiliproteins having antioxidant properties [98], while in green macroalgae (e.g., *U. intestinalis*), antioxidant pigments such as astaxanthin have been found [99]. 

### 3.6. Inhibition of Cancer Cell Growth 

**Figure 6:**
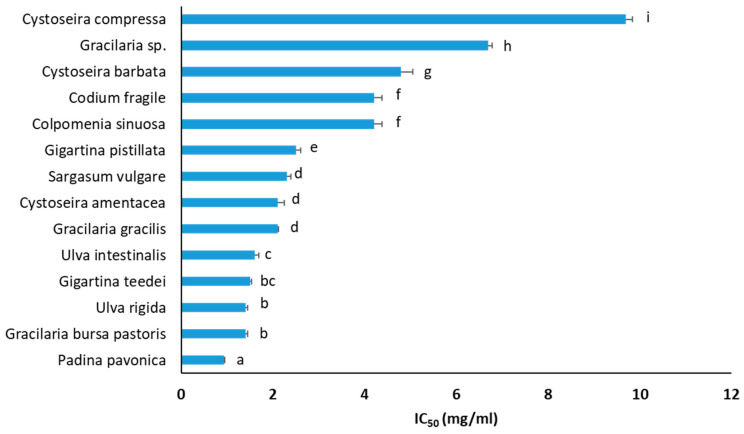
Inhibitory activity of macroalgae extracts against growth of liver cancer HepG2 cells. The IC_50_ values are presented as the mean ± SD from at least three independent experiments. All IC_50_ values are statistically significantly (*p* < 0.05) different compared to controls. Values having different letters are statistically different between them (*p* < 0.05).

Macroalgae extracts have been reported to possess anticancer activity [88]. Macroalgae’s main group of metabolites exhibiting anticancer properties are polysaccharides (e.g., sulfated polysaccharides and carrageenans), halogenated metabolites, phenols (e.g., bromophenols, polyphenols, and phlorotannins), pigments (e.g., pheophorbide A), iodine, lipids (e.g., sulfolipids), proteins (e.g., lectins), and terpenes (e.g., brominated terpenes and elatol) [88]. Thus, macroalgae extracts, apart from their antioxidant capacities, inhibited growth of liver HepG2 cancer cells (Figure 6). The macroalgae species’ IC_50_ values against HepG2 cell growth ranged from 0.93 to 9.70 mg/mL (Figure 6). The three macroalgae extracts that exhibited the highest inhibition against liver cancer cell proliferation were *P. pavonica* (IC_50_: 0.93 mg/mL), *U. rigida* (IC_50_: 1.40 mg/mL), and *G. bursa pastoris* (IC_50_: 1.40 mg/mL) (Figure 6). It should be noted that macroalgae species exhibiting high anticancer potential belong to all taxonomic groups, that is, Chlorophyta (e.g., *U. rigida* and *U. intestinalis*), Phaeophyceae (e.g., *P. pavonica*), and Rhodophyta (e.g., *G. bursa pastoris* and *G. teedei*). Importantly, *G. teedei* extract, demonstrating the highest antioxidant activity, was also included among the extracts having the greatest inhibition against the growth of cancer cells. This result indicated that the same compounds of *G. teedei* extract may account for both antioxidant and cancer cell growth inhibitory activities. 

Furthermore, according to our results, the macroalgae polyphenols’ roles in cancer cell growth inhibition were not clear, since there were extracts (e.g., *C. barbata*) having high TPCs and low inhibitory activity against cancer cell growth or vice versa (e.g., *G. bursa* pastoris) (Table 2, Figure 6). On the other hand, some extracts (e.g., *G. teedei* and *U. rigida*) with high TPC values exhibited also high inhibition against cancer cell growth (Table 2, Figure 6). Thus, it seems that for some macroalgae species, the total polyphenolic amount affects their anticancer potency, while there are also macroalgae species in which specific polyphenols may account for their anticancer activity and not their TPCs. Other studies have also reported, in agreement with us, that macroalgae extracts having high polyphenolic content exhibited low anticancer activity [100]. 

Furthermore, in accordance with our results, *P. pavonica*, a Phaeophyceae alga and the most potent extract, has been reported by others to inhibit HepG2 cells with an IC_50_ value (613 μg/mL) close to our value [101]. Moreover, *P. pavonica* extract was demonstrated to inhibit HCT-116 colon cancer cells [77] as well as osteosarcoma [55], lung, cervical, intestinal, larynx, and breast cancer cells [77] through molecular mechanisms such as apoptosis mediated by p53 protein [55]. In addition, El-Sheekh et al. [101] showed that *P. pavonica* decreased in vivo Ehrlich ascites carcinoma due to apoptosis. These *P. pavonica* activities were attributed mainly to its polysaccharides, sterols (e.g., fucosterol), terpenes (e.g., phytol), and fatty acids (e.g., palmitic acid) [55,101]. 

Some of the tested macroalgae species were reported previously to inhibit cancer cell growth. For example, extracts from the Phaeophyceae alga *C. barbata*, rich in phlorotannins, have been demonstrated to inhibit lung A549 [52,75], colon HT-29, breast MCF-7 [52], melanoma Fem-x, and chronic myelogenous leukemia K562 [75] cancer cells through increases in ROS, arrest at the subG1 phase, and apoptosis [52]. Furthermore, Kosanic et al. [75] showed *C. amentacea* to inhibit colon LS174 cancer cells. Like our findings, Kosanic et al. [75] reported that *C. amentacea* had better anticancer activity than *C. compressa* and *C. barbata*. *C. amentacea* has also been demonstrated to inhibit lung, melanoma, and myelogenous leukemia cancer cells [75]. Unlike our results, in two studies, *C. compressa* extracts did not inhibit liver Hep3B [27] and colon LS174 cancer cell growth [75], but the extracts have been used at lower concentrations (up to 50 and 200 μg/mL, respectively) than our extract. Interestingly, Guner et al. [27] collected *C. compressa* from the Aegean Sea (i.e., coast of Urla, Izmir, Turkey), but from a different region than ours. In addition, extracts from *C. sinuosa*, another Phaeophyceae alga, have been reported to inhibit HCT-116 colon cancer cell growth with IC_50_ values depending on the extraction method [100]. This activity was attributed mainly to the polysaccharides fucoidan and alginate and mediated through cell cycle arrest at the G1 phase, ROS increase, and apoptosis [89,100]. Additionally, sulfated polysaccharides such as fucan composed of fucose, galactose, xylose, glucuronic acid, and mannose from the Phaeophyceae alga S. vulgare have been demonstrated to inhibit cervical HeLa cancer cells [102]. 

Moreover, *C. fragile*, belonging to Chlorophyta, has been reported to possess compounds such as sulfated polysaccharides [103] and clerosterol [104], which inhibited in vitro and in vivo melanoma growth through cell cycle arrest at the G1 phase and apoptosis [104] as well as in vivo carcinoma metastasis [105]. In another study, sulfated polysaccharides from *C. fragile*, which were mainly linear homopolymers comprising ß-1.4-linked D-mannose residues, mediated anticancer immune responses through activation of NK cells, leading to an increase in cytotoxic mediators such as IFN-γ, IL-12, and CD69 overexpression [106]. Additionally, like us, Nazarudin et al. [107] reported that the Chlorophyte *U. intestinalis* inhibited growth of liver HepG2 cancer cells. In addition, *U. intestinalis* extract inhibited cervical cancer cells by autophagy induction through increases of p53, Bax, atg12, and p62 proteins [108]. Furthermore, lipid extracts from *U. rigida* exhibited inhibition of breast MDA-MB-231 cancer cells [109].

Finally, in agreement with our finding that the Rhodophyte *G. pistillata* inhibited colon cancer cell growth, carrageenans ι-, κ-, and λ- (i.e., sulphated polysaccharides) isolated from this species decreased cancer stem cell-enriched tumorspheres derived from colon SW620, SW480, and HCT116 cancer cell lines [110].

### 3.7. Correlation Analysis

Spearman’s correlation analysis was performed to find out if there was any association between macroalgae extracts’ activities as assessed in DPPH^.^, ABTS^•+^, ^•^OH, O_2_^•*−*^, RP, DNA plasmid strand cleavage, and XTT assays (Table 3).

The results showed that there were high and significant correlations between IC_50_ values of the DPPH^.^ assay and IC_50_ values of ABTS^•+^ (r = 0.825; p < 0.01), and RP_0.5AU_ values (r = 0.964; *p* < 0.01) (Table 3). Moreover, the IC_50_ values of the ABTS^•+^ assay were significantly and highly correlated with RP_0.5AU_ values (r = 0.789; p < 0.01) (Table 3). The significantly high correlation between values of DPPH^.^, ABTS^•+^, and RP assays suggested that the same macroalgae extracts’ antioxidant compounds may account simultaneously for these two radicals’ scavenging and reducing activity. In addition, DPPH^.^ and ABTS^•+^ assays are based on both hydrogen atom transfer (HAT) and single electron transfer (SET) mechanisms, while RP is a SET-based method [79]. Thus, the significantly high correlation between DPPH^.^ and ABTS^•+^ values with RP also indicated that most of the tested macroalgae species’ antioxidants acted mainly as SETs. Furthermore, the significantly moderate correlation between extracts’ values of the DNA plasmid breakage assay with values of DPPH^.^, ABTS^•+^, and RP assays (Table 3) suggested that some of the macroalgae extracts’ antioxidant compounds may account simultaneously for radical scavenging, reducing activity and preventing ROS-induced DNA damage. However, the absence of a high correlation between ^•^OH and O_2_^•−^ assays’ values with those of DPPH^.^, ABTS^•+^, and RP assays indicated that macroalgae’s compounds scavenging the former radicals were different from those scavenging the latter. 

There was also a significantly high anticorrelation between TPC values and IC_50_ values of DPPH (−0.737; *p* < 0.01), ABTS^•+^ (−0.789; *p* < 0.01), and DNA plasmid strand cleavage assays (−0.768; *p* < 0.01) (Table 3). Importantly, the significantly high anticorrelation between TPC and IC_50_ values of DPPH, ABTS^•+^, and DNA plasmid breakage suggested that polyphenols may play significant roles in the tested macroalgae extracts’ antioxidant activity, although macroalgae extracts’ TPC values were low. As mentioned above, several studies have demonstrated polyphenols to account for macroalgae’s antioxidant activity [50,52,71,75]. However, the absence of significant correlation between ^•^OH and O_2_^•−^ assays’ values and TPCs suggested that especially for these two radicals’ scavenging, either macroalgae’s polyphenols might not be important, or specific polyphenols might be important instead of TPC. Namely, although specific polyphenols with high antioxidant potency exist at low amounts in macroalgae extracts, they may be able to scavenge ^•^OH and O_2_^•−^ radicals.

Finally, the absence of significantly high correlation between XTT assay values and those of antioxidant assays indicated that different macroalgae’s compounds accounted for anticancer and antioxidant activity (Table 3). Furthermore, the absence of a significant correlation between TPC and XTT assay’s IC_50_ values (Table 3) indicated that in most tested macroalgae extracts, polyphenols were not important for macroalgae’s anticancer activity. As mentioned above, according to our findings, the association between macroalgae’s polyphenols and anticancer activity was not clear. 

### 3.8. Clustering of Macroalgae Extracts Based on their Activities with Dendrogram and PCA

**Figure 7:**
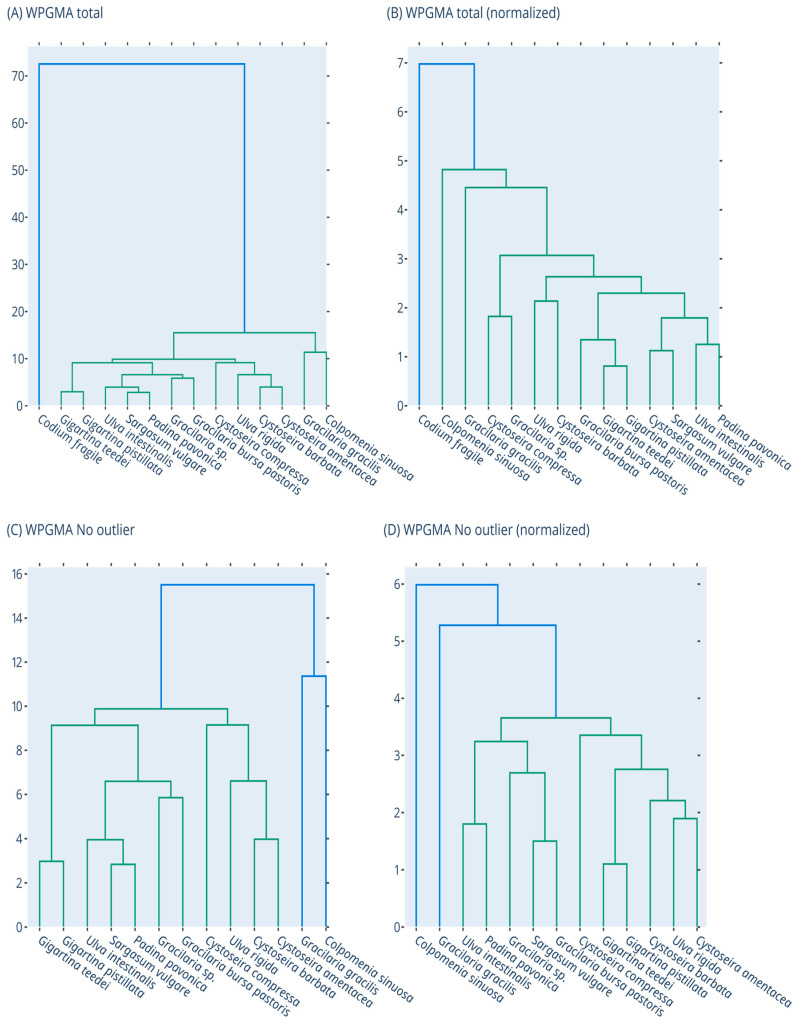
Dendrograms of the samples based on the measurements (raw and normalized) of 7 bioactivity metrics. All dendrograms were created using the WPGMA algorithm and the Euclidian distance. (**A**) Dendrogram of all the 14 samples based on raw measurements. (**B**) Dendrogram of the 14 samples after Z-score normalization. (**C**) Dendrogram of the 13 samples based on raw measurements after removing the outlier (*C. fragile*). (**D**) Dendrogram of the Z-score normalized data after removing the outlier (*C. fragile*).

**Figure 8:**
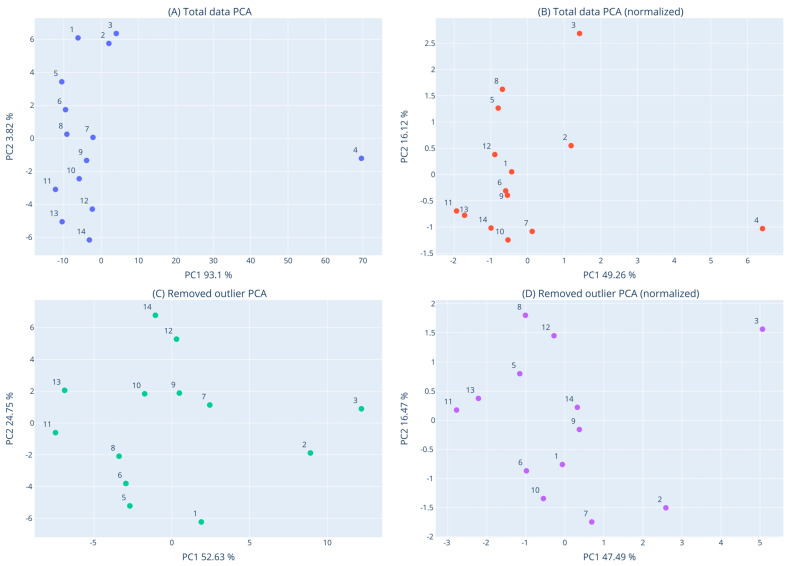
Principal component analysis (PCA) of the samples based on the 7 bioactivity metrics. The first 3 principal components are computed for each graph. Each dot represents one sample, and the number above it indicates the sample index. (**A**) PCA of all the 14 samples based on raw measurements. (**B**) PCA of the 14 sample, after Z-score normalization. (**C**) PCA of the 13 samples based on raw measurements after removing the outlier (*C. fragile*). (**D**) PCA of the Z-score normalized data after removing the outlier (*C. fragile*). The indexes of the various samples correspond to: 1: *U. rigida*, 2: *G. gracilis*, 3: *C. sinuosa*, 4: *C. fragile*, 5: *C. barbata*, 6: *C. amentacea*, 7: *U. intestinalis*, 8: *C. compressa*, 9: *S. vulgare*, 10: *P. pavonica*, 11: *G. teedei*, 12: *G*. sp., 13: *G. pistillata*, 14: *G. bursa pastoris*.

In order to detect similarities and differences among the tested macroalgae species (Supplementary Figure S2) in terms of their overall measured activities, dendrogram and PCA analysis were performed using the data from all the bioactivity assays (i.e., DPPH^.^, ABTS^•+^, ^•^OH, O_2_^•−^, RP, DNA plasmid strand cleavage, and XTT assay in HepG2 cells). The results of the dendrogram and PCA analysis are shown in Figure 7 and Figure 8. It is evident from both dendrogram and PCA analysis that the *C. fragile* extract was very different from the other thirteen samples and appeared as an outlier. This difference of *C. fragile* was due to its weak activity in all assays, especially the antioxidants. Reassuringly, other studies have also shown that *C. fragile* extracts had weak antioxidant activity compared to other macroalgae species [65]. As mentioned, *C. fragile* extract had also the least TPC value, probably accounting for its low antioxidant activity. 

Once the outlier was removed from the analyses, it was evident that the other 13 samples formed two major subclusters. The first one was composed of *G. gracilis* and *C. sinuosa*, whereas the second subcluster was composed of the other 11 species (Figure 7D). The clustering of *G. gracilis* and *C. sinuosa* was mainly attributed to their close potency order in DPPH^.^, ABTS^•+^, DNA plasmid strand cleavage, and RP assays. 

Moreover, *G. teedei* and *G. pistillata* extracts clustered together as sister groups (Figure 7D and Figure 8D). Indeed, these two Gigartina species were included among the most potent extracts in almost all assays, although the former had higher activity than the latter. All these suggested that *G. teedei* may contain similar bioactive compounds with G. pistillata, but in higher amounts. This conclusion was supported by the higher *G. teedei*’s polyphenolic amount compared to *G. pistillata*. 

Among Cystoseira species, *C. amentacea* and *C. barbata* clustered more closely compared to *C. compressa* (Figure 7D and Figure 8D). According to RACI values, *C. barbata* exhibited the best antioxidant activity, while it had also about a 2-fold higher TPC than the other two species. However, *C. amentacea* exhibited better anticancer activity than the other species. 

Furthermore, the three Gracilaria species’ extracts did not cluster together. Specifically, G. bursa pastoris and G. sp. clustered separately from *G. gracilis* (Figure 7D and Figure 8D). In antioxidant assays, the main differences between *G. gracilis* and G. bursa pastoris were exhibited in scavenging of ^•^OH and O_2_^•−^ radicals. Moreover, G. bursa pastoris was more potent in anticancer assay than *G. gracilis*.

The two extracts of *U. rigida* and *U. intestinalis* also did not cluster too closely (Figure 7D). The two Ulva species exhibited similar activity in most antioxidant assays, but *U. rigida* was more potent in DPPH and DNA plasmid strand cleavage assays compared to *U. intestinalis*. However, *U. intestinalis* had higher inhibitory activity against colon cancer cell growth than *U. rigida*. 

Overall, the clustering of the tested macroalgae species suggests that between species of the same genus there are common bioactive compounds accounting for their similarities in some assays, but they also contain compounds characteristic of each species, which differentiate their activity in other assays.

## 4. Conclusions

The results showed that the extract from the red macroalgae *G. teedei* was the most potent in all antioxidant assays, while it had also the highest TPC. Interestingly, another member of the *Gigartina* genus, *G. pistillata*, was the second most potent species in antioxidant activity, followed by *C. barbata*. Moreover, the results suggested that extracts’ polyphenols might play important roles for their antioxidant activity. In addition, extracts’ chemopreventive potential was also supported by their ability to inhibit liver HepG2 cancer cell growth. *P. pavonica*, *G. bursa pastoris*, and *G. teedei* extracts exhibited the three most potent inhibitory activities against liver cancer cells. To the best of our knowledge, the present study is the first demonstrating the antioxidant activity of *G. teedei*; the anticancer potential of *G. teedei*, *G. gracilis*, and *G. bursa pastoris*; the inhibitory activity of *G. pistillata, C. amentacea, C. compressa*, and *C. barbata* against liver cancer cells; protection from ROS-induced DNA damage of *G. teedei*, *G. pistillata*, *S. vulgare*, *G. gracilis, C. amentacea, C. sinuosa, C. fragile, C. compressa*, and *P. pavonica* extracts; and TPCs of *C. sinuosa*, *C. fragile*, *C. barbata*, and *G. bursa pastoris* extracts. Moreover, it is the first time to the best of our knowledge that the macroalgae species *C. amentacea*, *G. pistillata*, *G. gracilis*, *U. intestinalis*, *U. rigida*, *C. barbata*, *C. sinuosa*, *C. fragile*, and *C. compressa* collected from the Aegean Sea were examined for their antioxidant and/or anticancer activities. 

Of course, further research is needed to investigate in depth the most potent macroalgae extracts’ molecular mechanisms and bioactive compounds accounting for the antioxidant and anticancer activities in human cells and in vivo experiments. The elucidation of the macroalgae extracts’ molecular mechanisms and bioactive molecules is necessary in order to use them as either food supplements or additives in biofunctional foods with chemopreventive effects on human health.

## Figures and Tables

**Table 1:** Macroalgae species, stations of collection, and their taxonomic functional-form groups.

Species	Stations	Taxonomic Group (Phylum/Classis)	Functional-Form Group
*Codium fragile* subsp. *fragile* (Suringar) Hariot	1.1	Chlorophyta/Ulvophyceae	Coarsely-branched
*Ulva intestinalis* Linnaeus	1.1		Sheet
*Ulva rigida* C. Agardh	1.2		Sheet
*Colpomenia sinuosa* var. *peregrina* Sauvageau	1.1	Ochrophyta/Phaeophyceae	Coarsely-branched
*Cystoseira barbata* (Stackhouse) C. Agardh	1.1		Thick–leathery
*Cystoseira compressa (*Esper) Gerloff & Nizamuddin	2		Thick–leathery
*Cystoseira amentacea* C. Agardh Bory de Saint-Vincent var. *amantaceaPadina pavonica* (Linnaeus) Thivy	1.1 2		Thick–leathery Thick–leathery
*Sargassum vulgare* C. Agardh	2		Thick–leathery
*Gigartina pistillata* (S.G. Gmelin) Stackhouse	3	Rhodophyta/Florideophyceae	Coarsely-branched
*Gigartina teedei* (Mertens ex Roth) J.V. Lamouroux	3		Coarsely-branched
*Gracilaria bursa-pastoris* S.G.Gmelin) P.C. Silva *Gracilaria gracilis (*Stackhouse*)* C. Steentoft, L.M. Irvine & Farnham *Gracilaria s*p.	4 1.1 3		Thick–leathery Thick–leathery Thick–leathery

**Table 2:** Extraction yields of macroalgae extracts and their total polyphenols and IC_50_ values of scavenging activity against DPPH^•^, ABTS^•+^, OH^•^, and O_2_^•*−*^ radicals.

	IC_50_ (mg/mL)				mg GAE/g dw Extract	
	DPPH^•a^	ABTS^•+ a^	^•^OH ^a^	O_2_^•− a^	TPC ^c^	Extraction Yield ^b^ (%)
***Macroalgae species***						
***Green macroalgae***						
*Ulva rigida*	5.50 ± 0.20 *	0.95 ± 0.04 *	0.70 ± 0.01 *	ND	4.15 ± 0.16	18.0 ± 0.90
*Ulva intestinalis*	10.00 ± 0.11 *	0.98 ± 0.02 *	0.45 ± 0.03 *	ND	2.11 ± 0.07	24.0 ± 0.96
*Codium fragile*	79.00 ± 10.27 *	15.00 ± 0.60 *	1.70 ± 0.07 *	3.30 ± 0.26 *	0.55 ± 0.04	46.7 ± 3.74
***Red macroalgae***						
*Gracilaria gracilis*	13.50 ± 1.20 *	1.45 ± 0.12 *	0.65 ± 0.02 *	6.40 ± 0.80 *	3.01 ± 0.08	25.4 ± 0.51
*Gracilaria* sp.	10.00 ± 0.80 *	0.47 ± 0.03 *	1.10 ± 0.09 *	0.36 ± 0.03 *	3.16 ± 0.09	25.5 ± 1.53
*Gracilaria bursa pastoris*	9.20 ± 0.074 *	1.60 ± 0.060 *	1.50 ± 0.040 *	0.14 ± 0.012 *	2.00 ± 0.03	20.2 ± 1.82
*Gigartina teedei*	0.31 ± 0.006 *	0.02 ± 0.001 *	0.10 ± 0.007 *	0.05 ± 0.003 *	12.53 ± 0.88	20.3 ± 1.42
*Gigartina pistillata*	2.10 ± 0.126 *	0.16 ± 0.012 *	0.14 ± 0.011 *	0.07 ± 0.001 *	3.70 ± 0.20	18.9 ± 0.95
***Brown macroalgae***						
*Colpomenia sinuosa*	15.00 ± 1.80 *	2.5 ± 0.12 *	10 ± 0.30 *	1.10 ± 0.04 *	0.69 ± 0.02	19.7 ± 1.57
*Cystoseira barbata*	1.40 ± 0.07 *	0.43 ± 0.03 *	2.60 ± 0.18 *	1.20 ± 0.04 *	5.76 ± 0.15	22.4 ± 2.46
*Cystoseira amentacea*	2.5 ± 0.05 *	0.58 ± 0.05 *	0.16 ± 0.01 *	1.40 ± 0.11 *	2.54 ± 0.14	34.1 ± 1.36
*Cystoseira compressa*	2.90 ± 0.06 *	0.75 ± 0.03 *	1.40 ± 0.11 *	1.10 ± 0.10 *	2.93 ± 0.13	28.3 ± 2.26
*Sargasum vulgare*	8.20 ± 0.82 *	1.40 ± 0.07 *	1.30 ± 0.02 *	0.60 ± 0.05 *	2.53 ± 0.03	27.0 ± 0.81
*Padina pavonica*	6.50 ± 0.45 *	0.38 ± 0.03 *	0.40 ± 0.01 *	0.40 ± 0.04 *	2.77 ± 0.17	18.4 ± 1.29
***Positive control***						
*Ascorbic acid*	0.005 ± 0.0002 *	0.004 ± 0.0001 *	0.218 ± 0.013 *	ND	NT	NT

^a^ Values are the mean ± SD of at least three separate triplicate experiments. ^b^ Values are the mean ± SD of at least three separate experiments. ND: Not determined IC_50_ values (i.e., these extracts could not achieve 50% inhibition at the tested concentrations). NT: Not tested. ^c^ TPC: total polyphenolic content. * *p* < 0.05, indicates significant difference from the control values.

**Table 3:** Correlation coefficient (r) values estimated from correlation analysis between values of macroalgae extracts in DPPH^.^, ABTS^•+^, ^•^OH, O_2_^•−^, RP, DNA plasmid strand cleavage (DNA protection), XTT, and TPC assays.

Methods	ABTS^•+^	^•^OH	O_2_^•−^	RP	DNA Protection	XTT HepG2	TPC
DPPH^.^	0.825 **	0.471	0.283	0.964 **	0.685 **	0.099	−0.737 **
ABTS^•+^		0.653 *	0.361	0.789 **	0.607 *	0.077	−0.789 **
^•^OH			0.403	0.451	0.198	0.494	−0.442
O_2_^•−^				0.289	0.340	0.457	−0.284
RP					0.568 *	0.075	−0.697 **
DNA protection						−0.136	−0.768 **
XTT HepG2							−0.004

* *p* < 0.05, indicates significant difference from the control values. ** *p* < 0.01, indicates significant difference from the control values.

## Data Availability

The data presented in this study are available in the main text of this article.

## Supplementary Materials

The following supporting information can be downloaded at: https://www.mdpi.com/article/10.3390/foods12061310/s1, Figure S1: HPLC chromatograms of macroalgae extracts at 280 nm. (1) *Ulva rigida*, (2) *Gracilaria gracilis*, (3) *Colpomenia sinuosa*, (4) *Codium fragile*, (5) *Cystoseira barbata*, (7) *Cystoseira amentacea*, (8) *Ulva intestinalis*, (9) *Cystoseira compressa*, (10) *Sargasum vulgare*, (11) *Padina pavonica*, (13) *Gigartina teedei*, (14) *Gracilaria* sp, (15) *Gigartina pistillata*, (16) *Gracilaria bursa pastoris*. Figure S2: Photos of the tested macroalgae. Chlorophyta: *U. rigida, U. intestinalis, C. fragile*; Phaeophyceae: *C. sinuosa, P. pavonica, S. vulgare, C. barbata, C. compressa, C. amentacea*; Rhodophyta: *G. gracilis, G.* sp., *G. bursa pastoris, G. pistillata, G. teedei*.
