# Supplementary material for: Antioxidant Activity and Inhibition of Liver Cancer Cells’ Growth of Extracts from 14 Marine Macroalgae Species of the Mediterranean Sea
Source: Foods. 2023 Mar 19;12(6):1310. doi: 10.3390/foods12061310 (PMC10048654; doi:10.3390/foods12061310)
Supplement: Supplementary file 1 [file foods-12-01310-s001.zip › Supplement Figure S1.pdf]

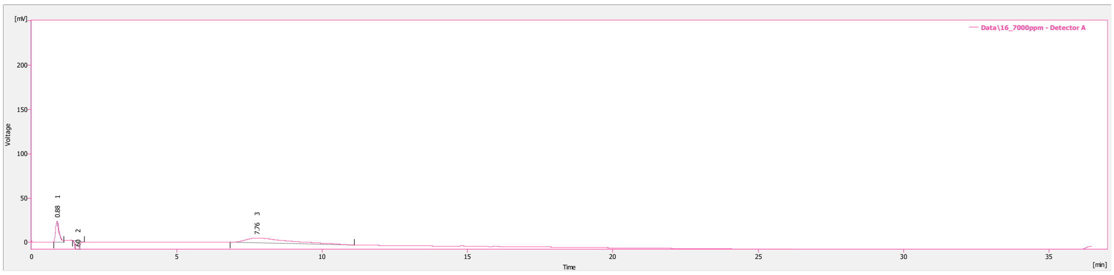

(16)

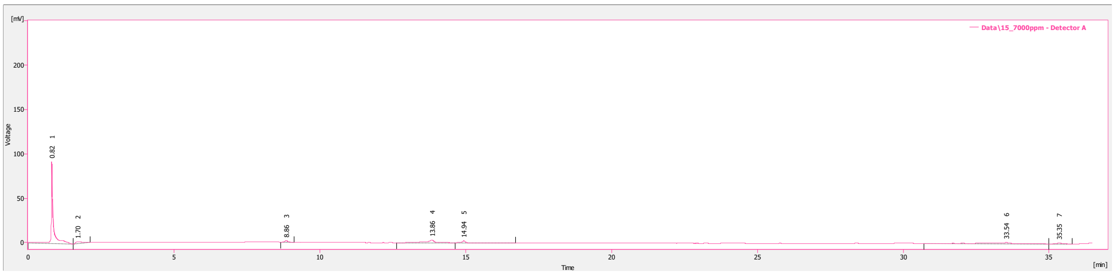

(15)

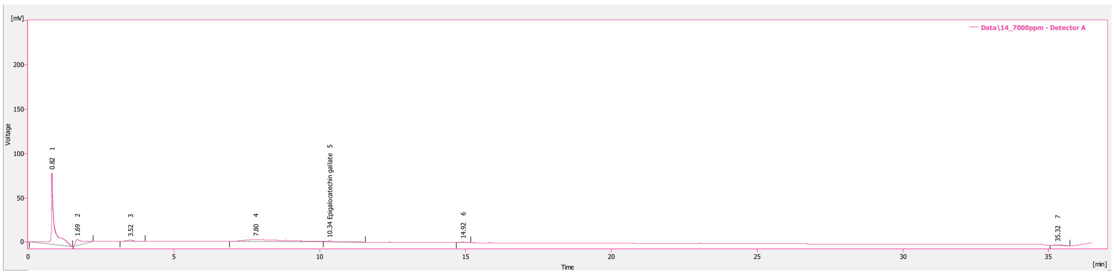

(14)

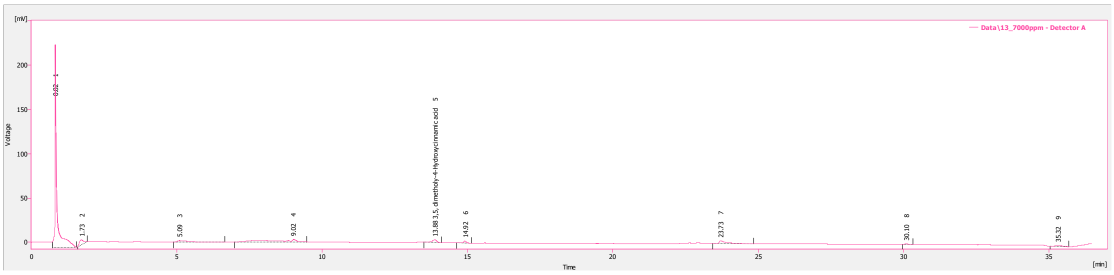

(13)

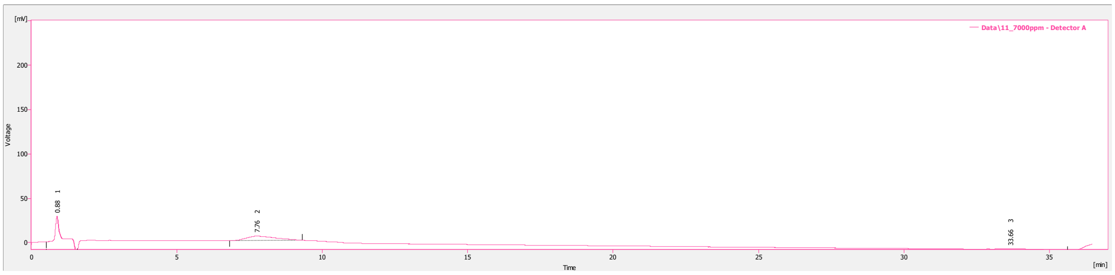

(11)

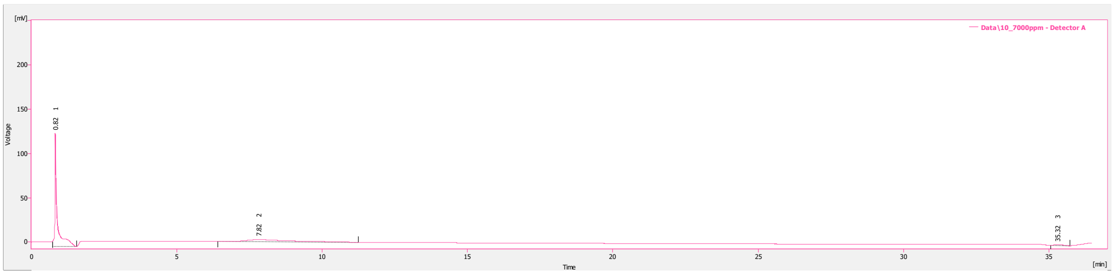

(10)

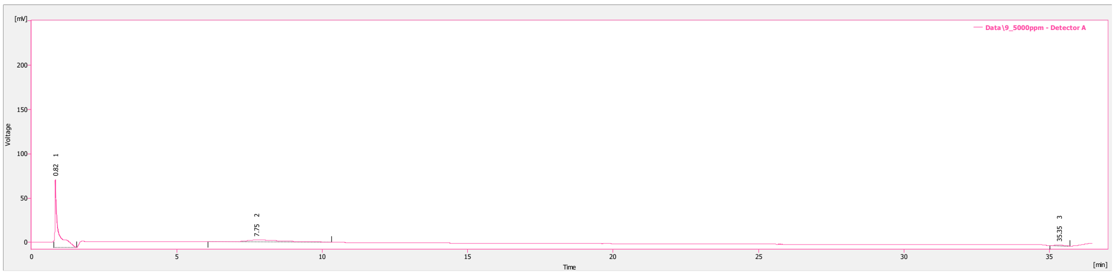

Data\9_5000ppm - Detector A
[mV]
Voltage
1 0.82
2 7.75
3 35.35
Time
[min]

(9)

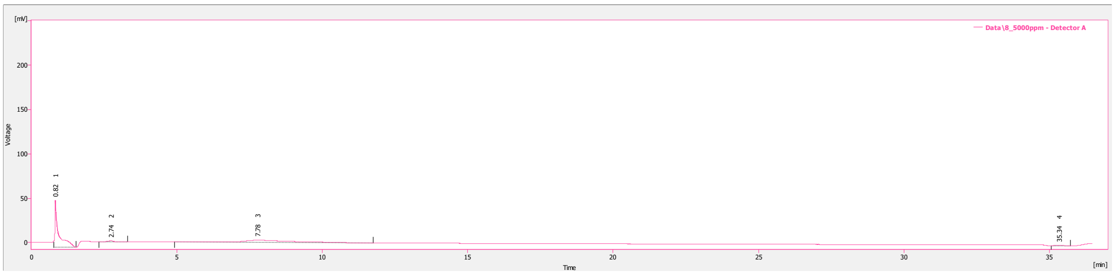

Data\8_5000ppm - Detector A
[mV]
Voltage
1 0.82
2 2.74
3 7.78
4 35.34
Time
[min]

(8)

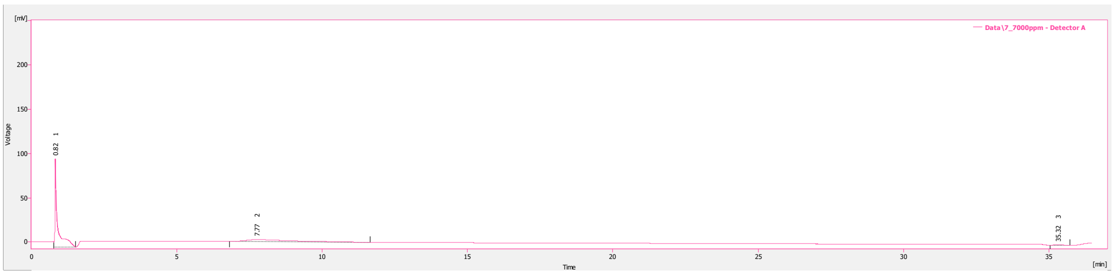

Data\7_7000ppm - Detector A
[mV]
Voltage
1 0.82
2 7.77
3 35.32
Time
[min]

(7)

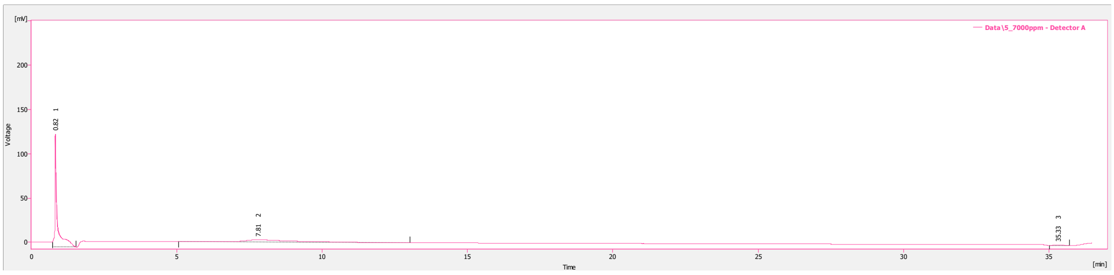

Data\5_7000ppm - Detector A
[mV]
Voltage
1 0.82
2 7.81
3 35.33
Time
[min]

(5)

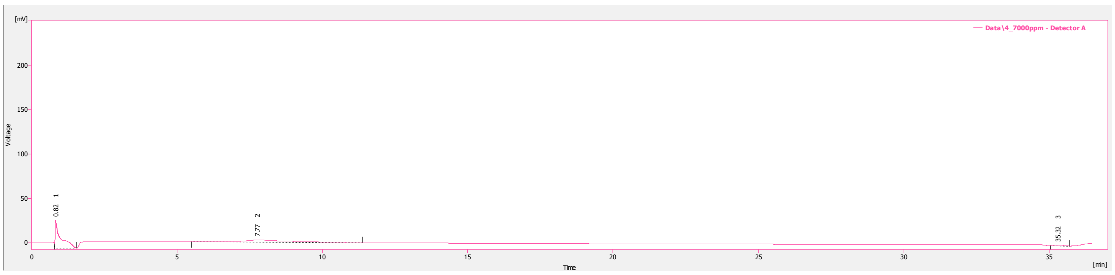

Data\4_7000ppm - Detector A
[mV]
Voltage
1 0.82
2 7.77
3 35.32
Time
[min]

(4)

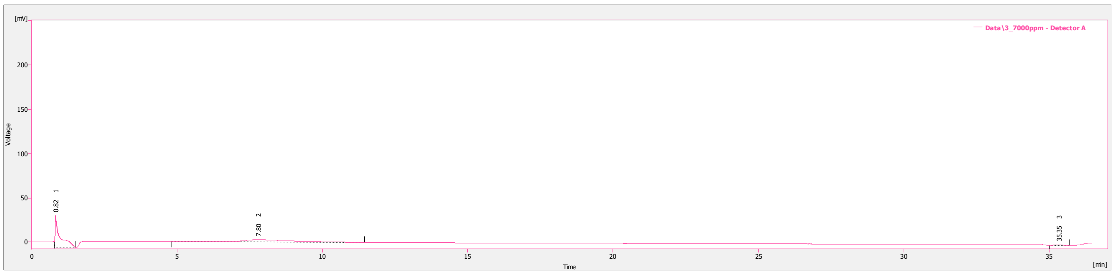

Data\3_7000ppm - Detector A
[mV]
Voltage
1 0.82
2 7.80
3 35.35
Time
[min]

(3)

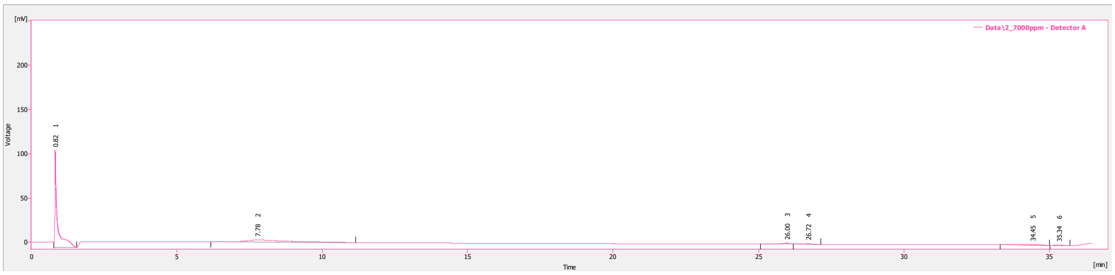

(2)

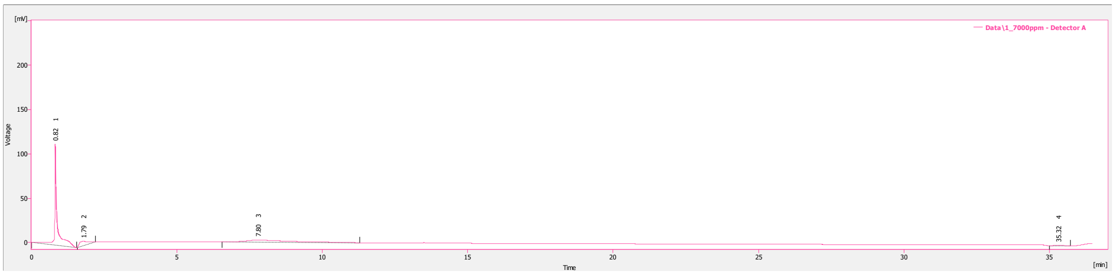

(1)

Figure S1. HPLC Chromatograms of macroalgae extracts at 280 nm. (1) *Ulva rigida*, (2) *Gracilaria gracilis*, (3) *Colpomenia sinuosa*, (4) *Codium fragile*, (5) *Cystoseira barbata*, (7) *Cystoseira amentacea*, (8) *Ulva intestinalis*, (9) *Cystoseira compressa*, (10) *Sargasum vulgare*, (11) *Padina pavonica,* (13) *Gigartina teedei*, (14) *Gracilaria* sp, (15) *Gigartina pistillata*, (16) *Gracilaria bursa pastoris*.
