# Supplementary material for: Antioxidant Activity and Inhibition of Liver Cancer Cells’ Growth of Extracts from 14 Marine Macroalgae Species of the Mediterranean Sea
Source: Foods. 2023 Mar 19;12(6):1310. doi: 10.3390/foods12061310 (PMC10048654; doi:10.3390/foods12061310)
Supplement: Supplementary file 1 [file foods-12-01310-s001.zip › Supplement Figure S2.pdf]

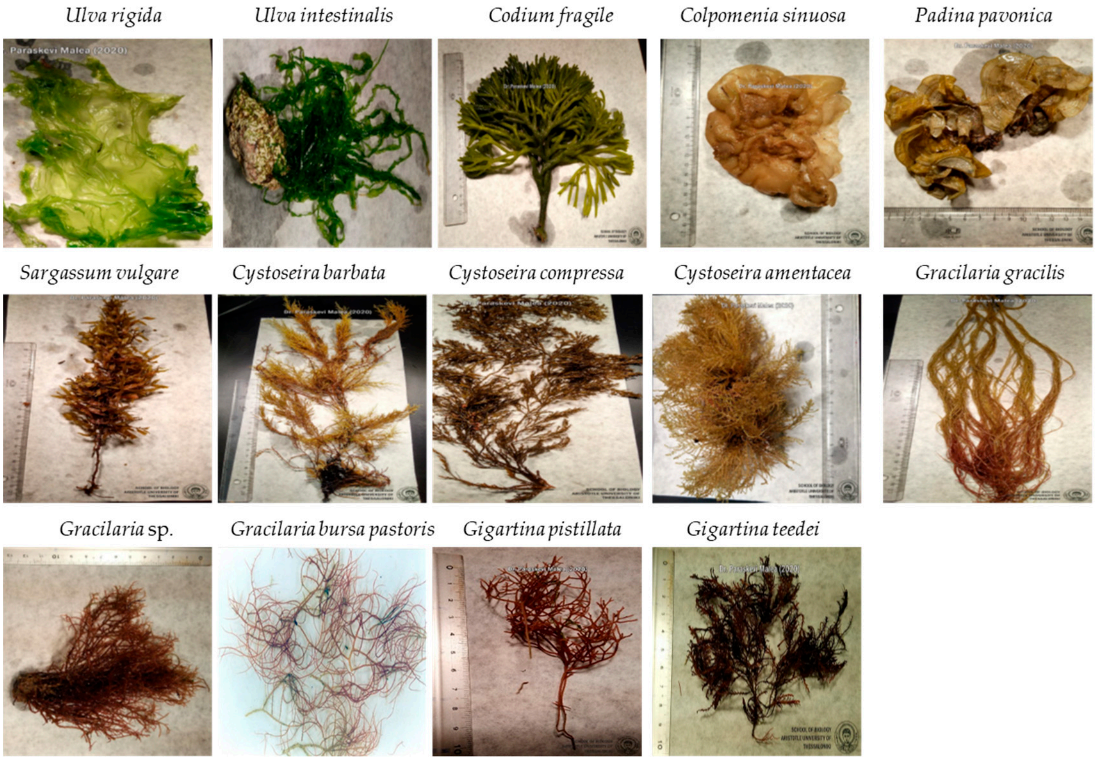

Figure S2. Photos of the tested macroalgae. Chlorophyta: *U. rigida, U. intestinalis, C. fragile;* Phaeophyceae*: C. sinuosa, P. pavonica, S. vulgare, C. barbata, C. compressa, C. amentacea;* Rhodophyta*: G. gracilis, G.* sp.*, G. bursa pastoris, G. pistillata, G. teedei*.
